# Full vibrational spectroscopy for simultaneous mechanical, structural and chemical analysis

**DOI:** 10.1038/s41467-026-74558-z

**Published:** 2026-07-07

**Authors:** Morteza Behrouzitabar, Kārlis Bērziņš, Renzo Vanna, Victor Alcolea-Rodriguez, Ben J. Boyd, Laura D’Alfonso, Cristian Manzoni, Dario Polli, Giulio Cerullo, Giuseppe Antonacci

**Affiliations:** 1 Department of Physics, University of Milano-Bicocca, Milano, Italy; 2 Specto Photonics, Milano, Italy; 3 Department of Pharmacy, Center for Pharmaceutical Data Science Education (CPDSE), University of Copenhagen, Copenhagen, Denmark; 4 CNR-Istituto di Fotonica e Nanotecnologie, CNR-IFN, Milano, Italy; 5 Dipartimento di Fisica, Politecnico di Milano, Milano, Italy; 6 Drug Delivery, Disposition and Dynamics, Monash Institute of Pharmaceutical Sciences, Parkville, VIC Australia; 7 Department of Pharmacy, University of Copenhagen, Copenhagen, Denmark

**Keywords:** Optics and photonics, Optical techniques

## Abstract

The light scattered inelastically from molecular vibrations occurring at different temporal and spatial scales carries intrinsic physical information about the illuminated sample. Mechanical, structural and chemical properties can be simultaneously retrieved by analyzing the full vibrational spectrum in different frequency ranges, yet strong elastic scattering near the laser frequency typically overwhelms the low-frequency region. Here, we present a method for measuring the full vibrational spectrum from 0.1 to  > 3,500 cm^−1^ using a Birefringence-Induced Phase Delay filter with high extinction ratio and ultranarrow bandwidth for efficient elastic suppression. Our multimodal, label-free approach enables the simultaneous acquisition of Brillouin, ultra-low-frequency Raman (ULFR), and Raman spectra from the same illumination voxel. We demonstrate the method acquiring Brillouin, ULFR, and Raman spectra from active pharmaceutical ingredients, revealing previously inaccessible distinctions in the amorphous forms of indomethacin and its mixtures with polyvinylpyrrolidone excipients. We further extended our approach to full vibrational imaging by acquiring maps of an ibuprofen tablet. Our findings demonstrate the high sensitivity and specificity of the multimodal platform to analyze chemical content while investigating mechanical and structural heterogeneities. Results open promising avenues in biomedical research and material science where key physical properties can be optically retrieved simultaneously at diffraction-limited spatial resolution.

## Abstract (web summary)

The authors report a method for measuring the full vibrational spectrum from 0.1 - 3500 cm-1 using a Birefringence-Induced Phase Delay filter. The approach opens avenues in biomedical research and material science, where physical properties can be optically retrieved simultaneously at diffraction-limited spatial resolution.

## Introduction

**Fig. 1:**
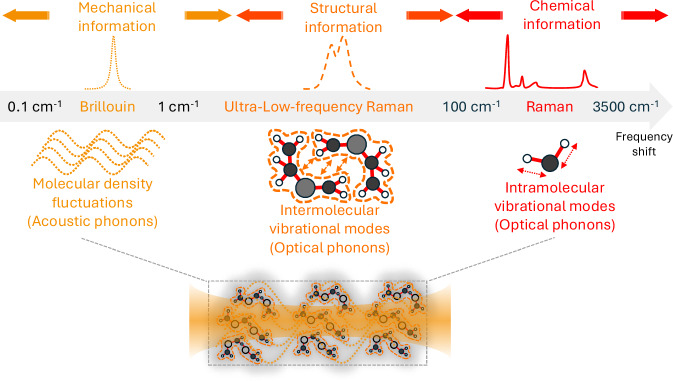
Full vibrational spectroscopy. Light interacts with various spontaneous vibrational states of matter across different frequency and spatial scales. The incident light loses (or gains) energy, resulting in broad light spectrum whose associated frequency shifts depend on whether the interaction occurs with molecular density fluctuations, intermolecular vibrational modes, or intramolecular vibrational modes. As a result, the full vibrational spectrum carries unique information about the material’s mechanical, structural, and chemical properties that can be retrieved by a non-contact, label-free, and three-dimensional optical approach.

Vibrational spectroscopy encompasses a broad set of techniques that probe the vibrational states of matter across different energy levels^1^. Among these techniques, we distinguish three complementary modalities of inelastic light scattering—Brillouin, ultra-low-frequency Raman (ULFR), and conventional Raman spectroscopy—each probing different vibrational regimes and providing distinct yet complementary information. Brillouin spectroscopy interrogates the interaction between light and acoustic phonons, which are spontaneous, thermally excited density fluctuations that carry information on the sample’s mechanical properties^2^. This results in frequency shifts on the order of ~0.1–1 cm^−1^, governed by ${\nu }_{B}=\pm (2n/\lambda )V\sin (\theta /2)$, where *ν*_*B*_ is the Brillouin frequency shift, *n* is the refractive index, *λ* the laser wavelength, *V* the acoustic velocity, and *θ* the scattering angle^3^. ULFR spectroscopy, also referred to as Raman THz spectroscopy, accesses collective intermolecular vibrations such as translations, librations, and skeletal deformations (optical phonons), which manifest as shifts in the 10–200 cm^−1^ range. These modes are highly sensitive to structural organization and phase, and unlike Brillouin scattering, ULFR spectra display a Stokes-anti-Stokes asymmetry dependent on thermal population distributions as described by the Boltzmann factor $\exp (-\hslash \omega /kT)$^4–6^. At higher frequency shifts (200–3500 cm^−1^), conventional Raman spectroscopy probes intramolecular vibrations of functional groups, offering chemically specific information. In particular, the 200–1800 cm^−1^ region, known as the Raman fingerprint region, and the 2800–3200 cm^−1^ CH-stretch region are widely used to identify molecular composition and structure in organic materials^7^. Together, these techniques enable a comprehensive vibrational analysis spanning mechanical, structural, and chemical domains (Fig. 1).

The all-optical, three-dimensional, and label-free nature of vibrational spectroscopy has driven its widespread adoption across disciplines such as mechanobiology, ophthalmology, and materials science. Brillouin spectroscopy has been used for 3D mechanical imaging^8–10^, clinical diagnostics^11,12^, and spin wave detection^13^, though its application to pharmaceutical systems remains limited due to detection challenges in turbid or solid-state media^14,15^. In contrast, ULFR spectroscopy has seen broader use in characterizing active pharmaceutical ingredients (API) solid-state forms^16–18^, layered materials^19–21^, and interlayer phonon dynamics. Conventional Raman spectroscopy remains widely employed across biomedicine^22,23^ and nanomaterials research^24^, and is firmly established in pharmaceutics for API quantification and solid-state analysis^25,26^.

While each of the aforementioned techniques provides unique information, combining data from multiple techniques has proven to be an effective approach to enhance both specificity and sensitivity, as well as to obtain more comprehensive insights. The combination of Brillouin and Raman techniques has been applied in various biomedical studies, including their use as a novel diagnostic tool for osteoarthritis^27^ and as a high-precision, multi-dimensional method for cancer cell analysis^28^. Combination of ULFR and Raman spectroscopy has been widely used in materials science for comprehensive analysis of interlayer interactions in two-dimensional and layered transition metal dichalcogenides^29^, as well as in pharmaceutics to detect intermediate transitional states during in situ crystallization in slurries^30^. Furthermore, independent Brillouin and ULFR studies on the polymorph characterization of APIs such as indomethacin (IND)^14,31^ indicate that simultaneous analysis of these spectral regimes may offer more valuable insights and enhance the analysis precision to differentiate mixture percentages as well as sample preparation history to characterize performance-critical attributes of pharmaceuticals.

Despite increasing interest in multimodal instrumentation, Brillouin and ULFR spectroscopies remain technically challenging due to the proximity of their weak spectral features to intense elastic background light from Rayleigh scattering and surface reflections. While multimodal microscopes combining Brillouin and high-frequency Raman spectra have been realized using edge-pass filters^32,33^, the purpose of such filters is not to provide simultaneous elastic background removal for Brillouin or ULFR spectroscopy. State-of-the-art detection relies on spectrometers with high spectral contrast or ultra-narrowband filters. Brillouin spectroscopy traditionally employed multi-pass Fabry–Pérot interferometers for their excellent resolution (~100 MHz) and contrast (~150 dB), though at the cost of long acquisition times (>10 s). Virtually Imaged Phased Array (VIPA)-based spectrometers^34^ have enabled faster acquisition (~100 ms)^35^, but suffer from limited contrast (~30 dB), which hinders performance in turbid samples. Contrast-enhancement strategies such as cross-dispersion^36^, field apodization^8,37^, and background deflection^38^ have improved VIPA contrast by up to ~60 dB. Other interferometric schemes offer ~40 dB suppression^39–42^, but often compromise robustness and throughput^43^. Moreover, Brillouin spectroscopy in combination with quantitative phase imaging has been employed for mechanical and structural analysis^44^. ULFR systems have evolved from low-throughput monochromators^45^ to Volume Bragg Grating (VBG) notch filters, enabling detection down to ~3–4 cm^−1^ with high throughput^46^, though their broad bandwidth makes them unsuitable for Brillouin. Hot rubidium vapor cells offer high suppression (~50 dB)^47,48^, but require laser locking and introduce spectral asymmetries. As a result, simultaneous acquisition of Brillouin, ULFR, and Raman spectra remains an open challenge.

Here, we introduce a method to acquire the full vibrational spectrum arising from inelastic scattered light for simultaneous interrogation of the mechanical, structural, and chemical properties of materials analyzed. Our method exploits a Birefringence-Induced Phase Delay (BIPD) filter to suppress the parasitic background light before parallel spectral detection of the Brillouin and Raman scattered light. In particular, the BIPD effectively rejects background signals regardless of their polarization state or physical origin. Independent of whether the background arises from Rayleigh or Mie scattering or from Fresnel reflections, the polarizer at the input of the BIPD imposes a linear polarization state on the incident light, thereby preserving the operating principle of the filter. We demonstrate the capabilities of this technique by acquiring the full inelastic spectrum of different pharmaceutical APIs, whose structural form critically influences their stability, solubility, and therapeutic performance^49^, yet remain poorly characterized as a consequence of their extreme turbidity. Using indomethacin as a test sample, our method revealed different vibrational spectral features of the same molecule in various solid-state forms. Specifically, our analysis demonstrated the sensitivity of the Brillouin regime to the amorphous and crystalline forms of indomethacin as well as to amorphous forms obtained with different thermal or processing histories. Further analysis was performed to demonstrate the sensitivity of this technique in differentiating mixture samples with varying indomethacin content. Additionally, we demonstrated the multimodal imaging capability of our system, acquiring spectral images of ibuprofen tablets, revealing mechanical and structural heterogeneity of ibuprofen content inside a tablet. Our findings demonstrate the enhanced specificity offered by the full vibrational spectroscopy and pave the way toward simultaneous characterization of the mechanical, structural, and chemical properties of a substance.

## Results

### Full vibrational confocal system

**Fig. 2:**
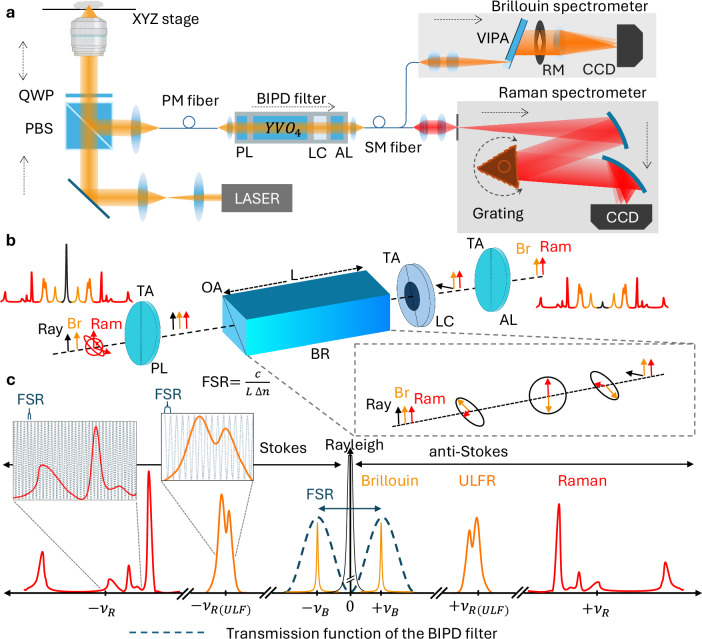
Multimodal Brillouin and Raman confocal system. **a** Schematic of the custom confocal multimodal Brillouin and Raman system. Backscattered elastic and inelastic signals are coupled into a single-mode polarization-maintaining (PM) fiber acting as a confocal pinhole. Light transmitted is filtered by the BIPD module. **b** This includes a polarizer (PL) aligned to the input polarization and a birefringent (BR) crystal. A liquid crystal (LC) retarder tunes the phase delay to ensure Rayleigh photons are polarized orthogonally to the analyzer’s (AL) transmission axis (TA). **c** The filter’s transmission is maximized at the expected Brillouin frequency with an appropriate choice of the FSR. This causes high-frequency modulation in ULFR and Raman regions, resulting in a nominal optical loss of 3 dB, but without loss in spectral information. The light transmitted by the filter is coupled to a single-mode (SM) fiber to deliver the signal to the spectrometers. Specifically, the Brillouin spectrometer consists of a single-stage VIPA etalon assisted with a rhomboidal mask (RM) for contrast enhancement, while the Raman spectrometer is a grating-based commercial unit. QWP quarter wave plate, PBS polarizing beamsplitter.

**Fig. 3:**
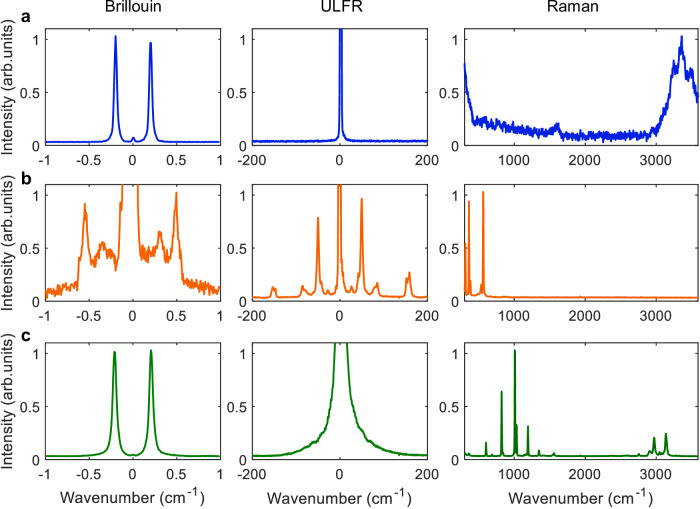
Full vibrational spectrum of standard calibration materials. **a** Water is used to calibrate the Brillouin spectral region, and representative spectra in ULFR and Raman regimes are demonstrated. Although the vibrational states of water are absent in the ULFR regime, characteristic H–O–H bending mode as well as symmetric and asymmetric O–H stretching modes in ~3000–3500 cm^−1^ are clearly visible. **b** *α*-Sulfur in crystalline form is used to calibrate the ULFR regime and representative peaks in Brillouin and Raman spectrum are shown. Notably, multiple peaks in the Brillouin domain suggest the existence of a heterogeneous mechanical structure, while sharp Raman peaks are observed in the region of 200–500 cm^−1^. **c** Finally, toluene is used to calibrate the Raman spectrum, and representative low-frequency vibrational modes are illustrated. The broad vibrational density of states (VDOS) feature in the ULFR regime stems from the intermolecular vibrational states. In the Brillouin and ULFR regimes, both Stokes and anti-Stokes peaks are shown around the central wavelength, whereas only the relevant Stokes side is shown in the high-frequency Raman region for better visualization.

Our multimodal spectroscopy system involves an inverted microscope where the sample is illuminated by a narrowband laser at the central wavelength of 660 nm (Fig. 2a). The backscattered light is collected and coupled to the BIPD filter to suppress unwanted elastic scattering background light^43^ and then analyzed by two separate spectrometer modules for parallel Brillouin and Raman spectral detection. The BIPD filter uses birefringent crystals to induce a relative phase delay between the incoming elastic and inelastic scattering signals, resulting in different polarization states at the output (Fig. 2b). A full-wave liquid crystal (LC) phase retarder allows a fine tuning of the phase delay to ensure a linear and orthogonal polarization state for the unwanted Rayleigh signal with respect to the transmission axis of an analyzer. This translates into a sinusoidal transmission function that is characterized by a free spectral range (FSR) given by FSR = *c*/LΔ*n* (Fig. 3c), where *L* is the length of the birefringent crystal and Δ*n* is the difference between ordinary and extraordinary refractive indexes. To remove the unwanted elastic (Rayleigh) background light while transmitting the inelastic light signal, we designed a filter with measured FSR of ~18.9 ± 0.2 GHz (0.63 ± 0.01 cm^−1^; Supplementary Fig. 1) so as to have high transmission over the expected Brillouin frequency range. On the other hand, since the selected FSR is much lower compared to the spectral resolution of conventional Raman spectrometers (typically 2–5 cm^−1^), the sinusoidal transmission profile results in a high-frequency modulation which is averaged out in both the low and the high-frequency Raman spectra, resulting in a nominal optical loss of 3 dB but without loss of relevant spectral information (Supplementary Fig. 2). To further support this, we shifted the BIPD transfer function by varying the LC voltage to verify that its high-frequency modulation response does not suppress Raman modes (Supplementary Fig. 3). After filtering, the Brillouin signal is analyzed using a single-stage, single-pass VIPA spectrometer combined with a rhomboidal diffraction mask to deflect the background light away from the dispersion axis^38^, achieving a measured spectral resolution and contrast of ~1 GHz and ~55 dB, respectively (Supplementary Fig. 4). In parallel, the whole Raman spectrum is acquired using a commercial Raman spectrometer equipped with diffraction gratings of 600 gr/mm and 2400 gr/mm, providing a measured spectral resolution of ~2.0 and ~1.3 cm^−1^, respectively. The two gratings are mounted on a rotating turret which allows their sequential use. The 2400 gr/mm grating is used to perform ULFR measurements with a measured spectral contrast of  ~50 dB (Supplementary Fig. 5). Notably, the spectral resolution defined by the grating corresponds to more than two times the modulation frequency defined by the FSR of the filter transmission function. Despite the optical losses, this condition ensures visibility across the entire vibrational spectrum without any noticeable loss of information. In this system, additional optical losses for the Raman signal arise because the polarization of scattered light depends on both the scattering mechanism and the excitation-detection geometry, leading to the rejection of certain components by the polarizing beamsplitter (PBS) before the input polarizer of the BIPD filter. For Rayleigh scattering, the polarization is largely preserved in isotropic scatterers, as it reflects the induced dipole moment. In Brillouin scattering, the polarization depends on the type of phonon involved, with longitudinal acoustic phonons, typical in 180^∘^ backscattering geometries, maintaining the incident polarization. Raman scattering exhibits more complex behavior, as the polarization of the scattered light is determined by the symmetry of the vibrational modes. Totally symmetric modes tend to preserve polarization, whereas non-totally symmetric modes show partial depolarization^50–52^. Because the polarization of scattered light depends on both sample properties and the measurement configuration, polarization selection can contribute to additional losses. To investigate the effect of polarization selection on Raman modes, we implemented an additional optical arm (Supplementary Fig. 6) to analyze the polarization state of the Raman vibrational modes of polystyrene using an analyzer. Results show that a loss of 2.3–7.4 dB is introduced due to polarization selection depending on the specific vibrational mode (Supplementary Fig. 7). To evaluate the high-frequency modulation losses predicted by simulations, we compared the Raman signal acquired with and without the BIPD filter in the detection path, finding a maximum measured loss of 7.3 dB in the fingerprint region around 1000 cm^−1^. Considering a 4 dB insertion loss caused by the absorption of the birefringent crystal and the fiber-coupling stages, the residual loss is close to the expected 3 dB loss arising from the intrinsic high-frequency modulation, with total losses reaching a maximum value of 10.2 dB in the C–H stretching region (Supplementary Fig. 8).

### Spectral calibration with reference materials

To demonstrate our method and validate the performance of our system, we first calibrated the instrumental dispersion axis along the different spectral regions. To this aim, we acquired the full vibrational spectrum of three well-characterized reference samples. Distilled water was selected as a reference material to calibrate the Brillouin spectral region below 1 cm^−1^ (Fig. 3a), given its well-known acoustic velocity (~1490 m/s). For the ULFR regime, we used crystalline *α*-sulfur (Fig. 3b), which exhibits four distinct major peaks in this spectral region. The measured peak positions of known frequency were used as a reference to calibrate the dispersion axis in units of wavenumber. Lastly, toluene was used to calibrate the Raman regime (Fig. 3c). We exploited toluene’s multiple high-intensity peaks to perform a linear transformation as a first approximation, considering the distinct peaks in the CH and fingerprint regions. However, more sophisticated calibration procedures can be applied to improve the calibration precision. Notably, the Rayleigh peak is absent in the Brillouin spectra of both water and toluene as a result of the intrinsic transparency of these two materials and the ability of our BIPD filter to suppress the Fresnel reflections arising along the optical path. By contrast, the high turbidity of crystalline sulfur leads to the unavoidable presence of the central Rayleigh peak. Nevertheless, it was still possible to observe the inelastic Brillouin and Raman spectra, demonstrating the effectiveness of our system in revealing the spectral signatures across the entire vibrational spectrum.

### Spectral investigation of APIs

**Fig. 4:**
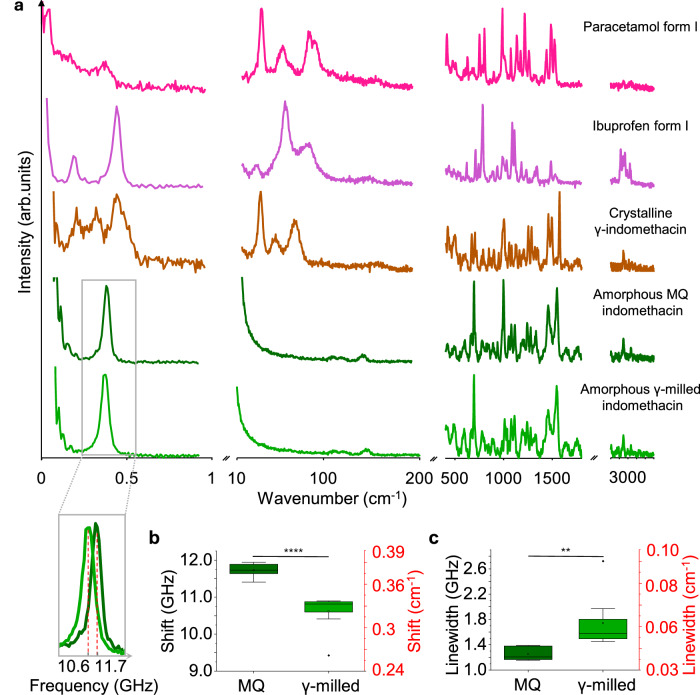
Full vibrational spectral analysis of APIs reveals amorphous sample history differentiation. **a** Full vibrational spectrum of reference APIs. Paracetamol form I, ibuprofen form I, crystalline *γ*-indomethacin, and amorphous *γ*-milled indomethacin exhibit distinctly different spectral signatures across all the three different spectral domains. Amorphous melt-quenched (MQ) and *γ*-milled indomethacin samples show similar features in the ULFR and Raman domains, while their respective Brillouin spectra manifest clear differences. **b** Indeed, a significantly higher (****p < 0.0001; *n* ≥ 5) Brillouin frequency shift was measured for the amorphous MQ indomethacin (11.73 ± 0.12 GHz) with respect to the amorphous *γ*-milled indomethacin (10.60 ± 0.51 GHz). **c** On the other hand, the Brillouin peak linewidth exhibited a significantly higher (**p < 0.01; *n* ≥ 5) linewidth for the amorphous *γ*-milled indomethacin (1.74 ± 0.43 GHz) with respect to the amorphous MQ indomethacin (1.25 ± 0.10 GHz).

We demonstrated the capability of our system to detect the full vibrational spectra of highly scattering APIs in both powder and tablet forms, enabling the simultaneous assessment of their mechanical, structural, and chemical properties. The first set of measurements was performed on common APIs, such as paracetamol, ibuprofen, and indomethacin, revealing noticeably different spectra across different spectral regions (Fig. 4a). These are widely used and extensively studied APIs, frequently employed as model systems in solid-state and formulation research. The scenarios addressed in this work reflect significant challenges in pharmaceutical development. For example, amorphous phases often lack clear structural fingerprints, making it difficult to demonstrate meaningful differences using conventional analytical techniques, despite their impact on stability, manufacturability, and bioavailability^53^. This issue is also relevant from a regulatory and intellectual property perspective. The sensitivity of the Brillouin scattering signal to viscoelastic variations, therefore, provides complementary information that is difficult to access with conventional Raman-based approaches alone. Notably, paracetamol form I and ibuprofen form I exhibited distinct Brillouin spectral features alongside their different and well-established ULFR and Raman signatures. These differences reflect the potential of this approach as a spectroscopy-based multidimensional platform for more effective and comprehensive pharmaceutical analysis, including solid-state characterization and classification of APIs. Such characterization is particularly important as different structural forms can significantly influence a drug’s solubility, stability, and therapeutic performance.

Differently prepared amorphous indomethacin phases provided an opportunity to probe vastly distinct short-range order environments. Herein, short-range order plays a critical role in dictating recrystallization behavior, which can be retrospectively assessed by analyzing the properties of the resulting crystalline products. This was illustrated by the preliminary differential scanning calorimetry results (Supplementary Fig. 9), where melt-quenched (MQ) and milled samples not only exhibited unique glass transition temperatures and thermal stability, but also were differentiated by recrystallization pathways. Crystalline *γ*-indomethacin, amorphous MQ *γ*-indomethacin, and ball-milled *γ*-indomethacin (*γ*-milled indomethacin) samples were specifically selected to investigate the capabilities of our full vibrational spectroscopy approach to offer a clearer differentiation between ordered and disordered states. Crystalline *γ*-indomethacin, MQ amorphous indomethacin, and ball-milled amorphous indomethacin were selected to represent distinct solid-state conditions of the same compound. While both MQ and ball-milled samples lack long-range order, they arise from fundamentally different preparation routes, which can lead to differences in local packing and molecular mobility. This distinction is relevant in the broader context of amorphous solid dispersions, which are now widely used formulation strategies and are produced through diverse processing routes, including melt-based and mechanically driven approaches. As different manufacturing pathways can yield amorphous materials with distinct physical properties, the ability to differentiate processing-dependent amorphous states is important for formulation development, stability assessment, and regulatory characterization^54^. The crystalline form exhibits multiple distinct peaks (lattice modes) while the amorphous form displays a broad vibrational density of states (VDOS) in the ULFR domain, as expected. On the other hand, the measured Brillouin spectra suggest a uniform mechanical structure for the amorphous form, exhibiting a single peak, while a more complex and heterogeneous mechanical structure is observed for the crystalline form, manifesting multiple peaks. Further analysis of the amorphous forms with different sample history, such as MQ and *γ*-milled indomethacin, highlights clear differences in the Brillouin spectra as opposite to the acquired ULFR and Raman spectral features, which manifest minimal differences that could only be detected by deeper spectral analysis (Supplementary Fig. 9). Analysis of the Brillouin spectrum of the different amorphous forms (Fig. 4b) revealed that the Brillouin shift of amorphous MQ indomethacin (11.73 ± 0.12 GHz) is significantly higher (*p* < 0.0001) than the amorphous *γ*-milled indomethacin (10.60 ± 0.51 GHz). Specifically, the higher frequency shift of the amorphous MQ indomethacin suggests a potentially “stiffer" environment compared to the amorphous *γ*-milled indomethacin. Furthermore, the Brillouin linewidth (Fig. 4c) of *γ*-milled indomethacin (1.74 ± 0.43 GHz) was significantly higher (*p* < 0.01) compared to the amorphous MQ (1.25 ± 0.10 GHz), where narrower linewidth indicates a longer relaxation time of the acoustic phonons, suggesting less damping and thus lower material viscosity. Overall, these results highlight the high sensitivity of the Brillouin spectral regime to differentiate amorphous samples with varying thermal or processing histories.

### Enhanced sensitivity of full vibrational spectroscopy to analyze API-excipient mixtures

**Fig. 5:**
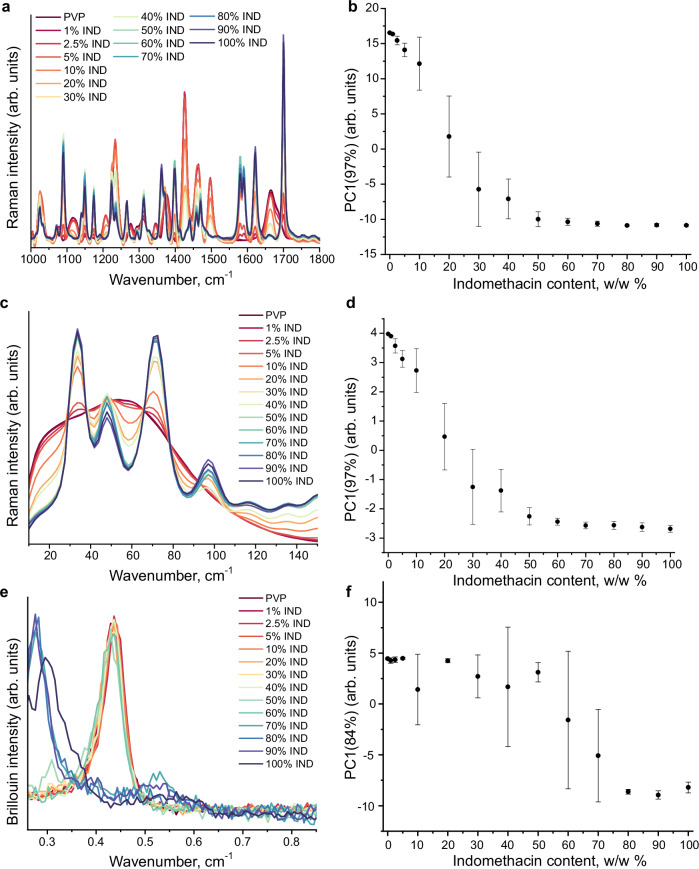
Spectral characterization of indomethacin-PVP mixtures. **a**, **c**, **e** Normalized Raman, ULFR, and Brillouin spectra examples for different indomethacin (IND) and PVP physical mixtures. **b**, **d**, **f** Mean PC1 scores plot (*n* = 5) for respective spectral region in relation to indomethacin content (loading plots in Supplementary Fig. 11). While PC1 scores of ULFR and Raman spectra show variations for low PVP concentration, PC1 scores associated to the Brillouin spectra show a higher sensitivity to mixture content variation above 50% as a consequence of the relative peak intensity changes of the Brillouin peaks associated to indomethacin and PVP.

We extended our study by analyzing various indomethacin-polyvinylpyrrolidone (PVP) mixtures to demonstrate the capability of this approach to differentiate API-excipient blends across a range of compositions as well as to investigate their full spectral properties and potential molecular interactions. Exploratory data from dedicated ULFR/Raman instrumentation was applied to develop a spectroscopic framework for understanding the system (Fig. 5a, c) and later used as a reference to verify the coherence with a few spectra acquired using the full vibrational system (Supplementary Fig. 10). Both finite and larger compositional variations were explored, where data analysis was aided by principal component analysis (PCA; Fig. 5b, d) that can help to identify patterns in complex data by transforming them into a set of uncorrelated principal components (PCs). Regardless of the spectral input used, PC1 accounted for 97% of the spectral variance, showing a near-linear change between 0 and 50% w/w indomethacin content. However, no significant change in the PC1 score values was observed at higher drug concentrations. This is likely a consequence of the intrinsically larger Raman cross-sections of indomethacin compared to PVP, which dominated the spectral signature. Furthermore, no spectral deviations suggestive of intermolecular interactions between the components were observed, either visually or through further PCA, at any of the drug-excipient ratios. We extended our analysis to the Brillouin domain using our full vibrational system. Results showed different trends revealing notable, non-linear variations among the same samples, indicating clear differences in their mechanical properties. The Brillouin spectrum of mixture samples acquired with our system demonstrates the existence of two main peaks associated with indomethacin (lower shift) and PVP (higher shift; Fig. 5e). The spectral analysis aided by PCA, reveals that PC1 (accounted for the 84% of the spectral variance) demonstrates lower and higher sensitivity to smaller and higher indomethacin content changes respectively, compared to the Raman and ULFR spectra (Fig. 5f). Loading plot of the Brillouin PC1 (Supplementary Fig. 11) shows the main spectral features constituting PC1 and from that it can be concluded that the altered ratio between the two Brillouin peaks is accounted for the variance in the scores of PC1 across the mixtures. Notably, the Brillouin spectrum associated with pure indomethacin shows variations in the frequency shift at higher concentration ratios, which potentially demonstrate the sensitivity of Brillouin light scattering to subtle changes in the crystalline architecture. We further performed concatenated spectral analysis by combining data from all spectral domains (Supplementary Fig. 12) and in pairwise combinations (Supplementary Fig. 13). The results clearly demonstrate the benefit of incorporating Brillouin data in the analysis. Combinations including Brillouin spectra exhibit a more extended and near-monotonic concentration-dependent trend in PC1, whereas the combination of just ULFR and Raman data shows earlier saturation at intermediate compositions.

These results demonstrate the increased sensitivity of the full vibrational approach to differentiate API-excipient blends and the complementarity of the three distinct spectral domains in providing unique information to distinguish different solid-state forms as well as physical mixtures for different contents.

### Full vibrational imaging

**Fig. 6:**
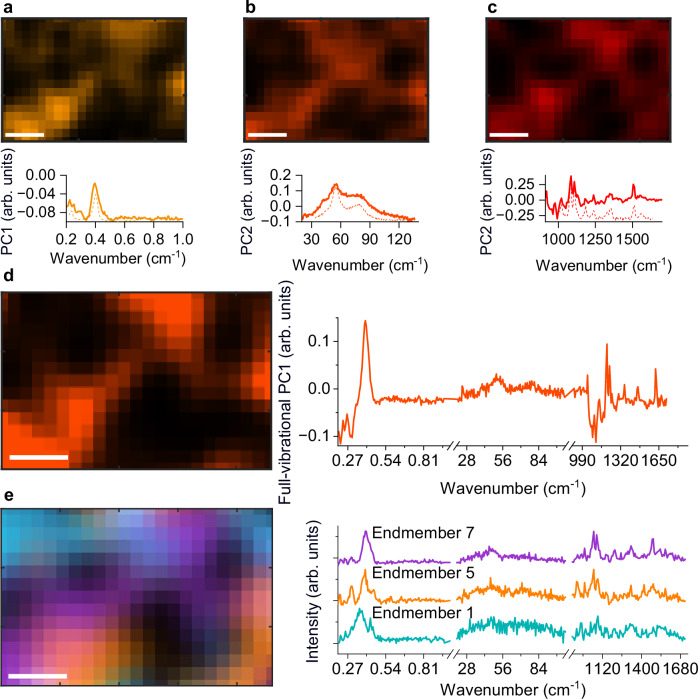
Full-vibrational spectral maps of an ibuprofen tablet. **a–c** Brillouin, ULFR, and Raman maps with respective loading plots associated with the relevant principal component (PC) show the mechanical, structural, and chemical distribution of ibuprofen across the same field of view of a reference tablet. Dashed lines represent measured spectral data of Ibuprofen. **d** Concatenated full vibrational spectrum analyzed with PCA exhibits spectral features through the entire domain, further enhancing the image contrast and compound specificity. **e** Endmembers identified using the N-FINDR algorithm revealed mechanical heterogeneities of ibuprofen across chemically homogeneous areas. Scale bar corresponds to 100 μm.

To demonstrate the unique capability of our system to perform parallel spectral imaging in the three distinct domains, we acquired Brillouin, ULFR, and Raman maps of ibuprofen tablets (racemic mixture with starch excipient) across the same region of interest of 320 × 480 μm^2^ with stepsize of 20 μm. Figure 6a–c present the results of a dedicated PCA conducted separately for each spectral region, based on the spectral references shown in Fig. 4a. The loading plots associated with the most significant PCs in each region highlight spectral features characteristic of ibuprofen. Other PCs are reported in Supplementary Fig. 14. The visual similarity across the resulting maps reflects the spatial distribution and relative concentration of ibuprofen, effectively captured through the full vibrational spectrum. Notably, the acquired spectral maps reveal not only the chemical distribution of ibuprofen within the tablet, distinguishing it from the starch excipient, but also provide insight into its underlying mechanical and structural heterogeneity. We extended our analysis by performing PCA over the full vibrational spectrum, which is obtained by concatenating the three spectral regimes (Fig. 6d). Spectral features from three spectral regions contribute to the PC1, significantly enhancing the specificity to investigate the ibuprofen distribution and content. The similarity between the individual PCA maps is expected, given that each spectral domain responds to the presence of a specific material. This validation is important because it allows us to confirm that each spectral domain can distinguish Ibuprofen from starch. However, despite the overall similarity, there are subtle differences among the maps. These differences mainly occur at the border of the region, indicating the presence of Ibuprofen, leading to slight variations in the observed pattern. To better understand the subtle differences between the PCA maps, we performed further analysis using the N-FINDR algorithm.

Finally, the endmembers extracted using the N-FINDR algorithm to analyze the mechanical heterogeneity within the sample (Fig. 6e). In the N-FINDR algorithm, endmembers are selected directly from the hyperspectral dataset as spectra associated with individual image pixels. Specifically, the method identifies the set of pixel spectra that maximizes the volume of the simplex enclosing the data in spectral space, such that the resulting endmembers correspond to real measured spectra rather than synthetic or averaged components^55^. In our analysis, endmember 1 exhibits a Brillouin peak at a lower frequency shift, which can be primarily attributed to regions of reduced stiffness, potentially corresponding to areas with higher excipient concentration as inferred from ULFR and Raman spectral features. Although endmembers 7 and 5 display similar signatures in the ULFR and Raman regions—suggesting the presence of ibuprofen—they reveal distinct spectral characteristics in their respective Brillouin spectra, where endmember 5 exhibits an additional resolved Brillouin peak at ~0.45 cm^−1^ (~13.5 GHz). These findings demonstrate that full vibrational imaging not only enables chemical identification but also allows the detection of mechanical variations within the same compound in a complex mixture.

## Discussion

In this work, we introduced an all-optical, non-destructive, and label-free method to acquire the full vibrational spectrum of a material, from 0.1 to > 3500 cm^−1^, to simultaneously determine its mechanical, structural, and chemical properties. While the integration of standard Raman spectroscopy systems with either Brillouin or ULFR modalities has been previously demonstrated individually, the development of an extended multimodal system capable of probing the full vibrational spectrum in all three main spectral windows has remained a major challenge as a consequence of the strong elastic background light, which typically overwhelms the nearby Brillouin and ULFR spectral signatures. Here, we used a BIPD filter with an appropriate choice of the FSR to effectively reject the unwanted elastic background light across the entire vibrational spectrum. In turn, our system was capable of simultaneously retrieving the inelastic Brillouin, ULFR and Raman spectra originating from the same illumination voxel.

We demonstrated our method to analyze solid-state forms of APIs. Assessment of the solid-state form of APIs is crucial during the production process, as properties such as solubility and processability directly affect bioavailability^56,57^. The introduction of Brillouin spectroscopy, in addition to previously used ULFR and Raman techniques in solid-state form analysis of APIs, provides deeper understanding as well as a higher sensitivity and selectivity by assessing their underlying viscoelastic properties. Access to a wider spectral dataset indeed enhances the differentiation of structural API configurations, with each spectral domain uniquely illustrating their distinctive features. This aspect is particularly important for the characterization of amorphous pharmaceutical systems, especially given the growing interest in so-called “polyamorphs” and the resulting need for reliable tools to differentiate them for both research and regulatory purposes. Our results on differently prepared amorphous indomethacin phases demonstrate the potential of the Brillouin regime to distinguish between them based on their respective peak characteristics, where simultaneous access to multimodal information could further support and refine this differentiation. Similarly, the analysis of indomethacin and PVP blends at varying ratios—encompassing both crystalline and amorphous compositions—demonstrates the distinct sensitivity of each spectral domain to particular material characteristics. Notably, the technique is capable of distinguishing between amorphous samples with varying preparation conditions—an aspect that is often challenging to resolve using conventional analytical methods. Our findings shown that the variance in the Brillouin peaks of different indomethacin-PVP mixtures provides a more sensitive analysis of these compounds at higher mixture concentrations compared to Raman and ULFR spectroscopy. Such spectral differentiation may be strategically leveraged in pharmaceutical applications, where detailed structural insight is critical for the rational design and manufacturing control of solid dosage forms and related systems.

We further showcase the versatility of our full vibrational system, demonstrating its extension as a new multimodal and label-free imaging method. The fully acquired vibrational spectral maps of an ibuprofen tablet showed improved accuracy in determining the microscale spatial distribution of the ibuprofen, which showed significantly different spectral characteristics compared to the excipient in all distinct spectral regions. Importantly, we showed that our full vibrational imaging method outperforms analyses based on individual spectral methods while providing fundamental insights about the mechanical and structural heterogeneity of specific components.

Our results open up exciting new opportunities for the pharmaceutical industry, where a comprehensive vibrational system can play a pivotal role not only in drug discovery but also in real-time, quantitative characterization during pharmaceutical manufacturing and quality control with minimal sample waste. Beyond pharmaceuticals, our full vibrational system has strong potential for applications in life sciences and materials science, offering unique and complementary physical insights into the samples under analysis with unprecedented sensitivity and specificity. Recent studies have demonstrated that vibrational spectroscopy can provide complementary insights into nuclear organization in living cells. Low-frequency Raman imaging has been shown to reveal information about nucleic acid packing and ordering, enabling visualization of DNA compaction in the nucleus without labeling^58^. In parallel, combined Raman-Brillouin microscopy has been used to quantify both the biochemical composition and viscoelastic properties of chromatin, revealing differences between euchromatin and heterochromatin and highlighting the role of molecular constituents such as lipids in determining chromatin mechanics^59^. In this context, a system capable of simultaneously measuring Brillouin, ULFR, and Raman spectra could provide complementary mechanical, structural, and chemical information within the same measurement. Such multimodal characterization may therefore offer a useful approach for studying chromatin organization and nuclear architecture in living cells by linking molecular composition, structural ordering, and mechanical properties within a unified experimental framework. Ultimately, the integration of our vibrational approach with confocal microscopy paves the way for a fully optical, label-free, multimodal imaging technique capable of co-registering three-dimensional mechanical, structural, and chemical maps with sub-micron spatial resolution.

## Methods

### Full vibrational system

A single-longitudinal-mode laser (Cobolt AB, Flamenco) operating at *λ* = 660 nm enters into an inverted microscope (Olympus, IX73) and is focused on the sample by a 60× water-immersion objective lens (Olympus UPlanSApo 60×/1.20). The sample is placed on a quartz coverslip. The scattered light is collected by the same objective. The collected elastically and inelastically scattered light passes through a quarter wave plate and is reflected by a PBS to be coupled into the polarization-maintaining single-mode fiber, providing confocality and flexible beam delivery to the filter. Light collimated at the output of the fiber is transmitted through the BIPD filter and coupled back into a second single-mode fiber, delivering the Brillouin and Raman signals to the spectrometer units. The Brillouin spectrometer consists of a VIPA etalon of FSR = 60 GHz (LightMachinery)—equivalent to 2 cm^−1^—where light is focused by a cylindrical lens. A Fourier lens translates the angular content of the output interference fringes into the spatial domain to be acquired by a CCD camera (Retiga R1). A rhomboidal mask is used right before the lens to deflect the Rayleigh signal away from the dispersion axis of the VIPA to obtain an improved spectral contrast. The Raman spectrometer is a commercial Isoplane160 (Princeton Instruments) spectrometer equipped with a rotating turret holding two gratings of 600 gr/mm and 2400 gr/mm, which provides automated switching between the two gratings, is connected to a front-illuminated CCD camera (PIXIS256F, Princeton Instruments).

### BIPD filter module

A birefringent YVO_4_ crystal of 60 mm length is mounted between two Glan–Taylor polarizers. While the output polarizer (analyzer) is needed to reject the Rayleigh component, the input polarizer is used to arrange the input polarization state with respect to the optical axis of the YVO_4_ crystal. The latter is on a plane parallel to the input/output surfaces, and is rotated by 45° with respect to the transmission axes of the input and output polarizers. The input/output surfaces of the crystal have anti-reflection coatings. The FSR of the filter is measured ~0.63 cm^−1^, ensuring maximum transmission at the expected Brillouin shift.

### Acquisition and data processing

Brillouin, ULR, and Raman spectra were collected sequentially by switching the optical fiber between spectrometer units and automatically changing the dispersion gratings with exposure time of 5 s/spectrum (Brillouin) and 60 s/spectrum (Raman) with a laser excitation power of 8 mW at the sample plane. Raw Brillouin spectral data were processed by fitting the intensity profiles using Lorentzian functions with a custom developed Matlab code and calibrating the dispersion axis with the spectral reference of distilled water. The spectral pre-processing of the Brillouin data for PCA analysis consists of spectral normalization (correction for laser intensity fluctuations), spectral shift compensation (for laser frequency drift), and finally removal of residual Rayleigh peak^60^. Spectral axis calibration of ULFR data (2400 gr/mm) was performed using the interpolation of well-known peaks of *α*-sulfur. Pre-processing of Raman data consists of Rubberband baseline-removal and subtraction of reference background spectrum with stray light from the spectrum. Linear conversion was used to calibrate the Raman spectral axis (600 gr/mm) using the peaks of toluene on the right and left sides of the spectrum, while more suitable calibration procedures can be implemented to increase the calibration precision.

### Materials

Indomethacin (2-1-[(4-chlorophenyl)carbonyl]-5-methoxy-2-methyl-1H-indol-3-ylacetic acid) (> 98.0%; Mw = 357.79 g/mol) was purchased from TCI (Zwijndrecht, Belgium), whereas PVP (Mw = ~360,000 g/mol) was purchased from Sigma-Aldrich (Søborg, Denmark). Ibuprofen ((RS)-2-(4-(2-methylpropyl)phenyl)propanoic acid) and paracetamol (N-(4-hydroxyphenyl)acetamide) were purchased from Angelini (Rome, Italy) and Bayer (Leverkusen, Germany), respectively. Sulfur rock (native sulfur) was used to obtain sulfur spectrum. Ibuprofen tablets containing 200 mg of ibuprofen and a starch-based excipient (for imaging purposes) were obtained from Farmacia Dott. Ambreck (Milan, Italy).

### Preparation of amorphous indomethacin samples

Amorphous indomethacin samples with different history (i.e., ball-milled and MQ) were prepared using adapted methods from the literature^61^: (i) 150 mg of indomethacin *γ*-form was ball-milled for 4 h (with 10 min hourly breaks) at a frequency of 30 Hz using a mixer mill MM 400 (Retsch GmbH, Haan, Germany) and 5 mL stainless steel jars containing one stainless steel ball with a diameter of 8 mm; (ii) 150 mg of indomethacin was MQ by heating the sample placed in an aluminum foil pan to 180 °C, holding it at the temperature for 3 min and cooling it to ambient temperature by placing the pan on top of aircooled stainless steel block for 10 min. The resulting glass film was gently ground using a mortar and pestle, and stored at −20 °C under reduced relative humidity (<5%).

### Preparation of physical mixtures

100 mg indomethacin *γ*-form / PVP samples containing 0, 1, 2.5, 5, 10, 20, 30, 40, 50, 60, 70, 80, 90% w/w of drug were mixed using a Hauschild centrifugal SpeedMixer*Ⓡ* (Hamm, Germany) inside 10 mL closed plastic containers. The samples were mixed at 1000 rpm for 5 min. These parameters were optimized from preliminary experiments to limit the potential molecular mixing between the components^62^.

### Preliminary Raman analysis of amorphous samples and physical mixtures

A THz-Raman*Ⓡ* system (Ondax Inc., Monrovia, CA, USA) equipped with a 300 mW, 785 nm laser was used for exploratory measurements of the prepared indomethacin samples prior to full vibrational analysis with the developed confocal microscope. Measurements were performed in a modular free-space optical configuration with a focal distance of ~8 mm and a laser spot size of 0.15–0.25 mm. Backscattered light was filtered using VBGs and detected with a CCD-equipped spectrograph (Andor iVac 316, Oxford Instruments, Abingdon, UK). Spectra were acquired over −870 to 3200 cm^−1^ with 4–6 cm^−1^ spectral resolution, averaging 60 scans at 0.25 s integration time.

Spectral preprocessing included cosmic spike removal, baseline correction, and standard normal variate normalization. Linear baseline correction was applied to the ULFR region (−300 to 300 cm^−1^), while a rubberband correction was used for the mid-frequency Raman region (1000–1800 cm^−1^). PCA was performed using Orange Data Mining 3.37.0 (University of Ljubljana, Ljubljana, Slovenia). For concatenated analysis of Brillouin, ULFR, and Raman data, each spectral block was independently preprocessed and normalized using Frobenius-norm block scaling prior to concatenation and PCA.

### Differential scanning calorimetry (DSC)

A differential scanning calorimeter (Q2000, TA Instruments, New Castle, DE, USA) equipped with a refrigerated cooling accessory was used. The instrument was calibrated with an indium standard. About 2–3 mg of sample was typically placed in a sealed and manually pinholed aluminum pan (TA Instruments, New Castle, DE, USA) for the analysis. The samples were heated from 20 to 200 °C using a 10 °C/min heating rate. The data analysis was carried out using the TRIOS 5.1.1 software package (TA Instruments, New Castle, DE, USA).

### Data processing for imaging modality

The full vibrational spectrum was obtained by pixel-wise concatenating of Brillouin, ULFR, and Raman spectral data into a single, continuous spectrum that spans the entire vibrational range. Prior to concatenating the spectrum, all spectra from the three modalities were min-max normalized to minimize the impact of relative intensity variations across the dataset. Subsequently, multivariate analysis was performed on the stitched spectral map using the RamApp platform (https://ramapp.io/). PCA was first applied, and the first principal component (PC1) was calibrated post hoc for improved interpretability. Additionally, N-FINDR algorithm was implemented. N-FINDR identified seven spectrally pure components, known as endmembers, which represent the most distinct spectral signatures within the dataset. Endmembers 1, 5, and 7 were selected based on their mechanical, structural, and chemical relevance.

## Supplementary information

Supplementary Information

Transparent Peer Review file

## Data Availability

The data that support the findings of this study are available from the corresponding authors on request.
