# Supplementary material for: Full vibrational spectroscopy for simultaneous mechanical, structural and chemical analysis
Source: Nat Commun. 2026 Jul 7;17:5632. doi: 10.1038/s41467-026-74558-z (PMC13342256; doi:10.1038/s41467-026-74558-z)
Supplement: Supplementary file 1 — Supplementary Information [file 41467_2026_74558_MOESM1_ESM.pdf]

Full vibrational spectroscopy for simultaneous mechanical, structural and chemical analysis

Morteza Behrouzitabar[1,2*], Karlis Berzins[3], Renzo Vanna[4], Victor Alcolea-Rodriguez[4,5], Ben J. Boyd[6,7], Laura D'Alfonso[1], Cristian Manzoni[4], Dario Polli[2,4,5], Giulio Cerullo[4,5], Giuseppe Antonacci[2**]

[1] Department of physics, University of Milano-Bicocca,Piazza della Scienza 3, 20126 Milano, Italy
[2] Specto Photonics, Via Giulio e Corrado Venini 18, 20127 Milano, Italy
[3] Department of Pharmacy, University of Copenhagen, Universitetsparken 2, 2100 Copenhagen, Denmark
[4] CNR-Istituto di Fotonica e Nanotecnologie, CNR-IFN, Piazza Leonardo da Vinci 32, 20133 Milano, Italy
[5] Dipartimento di Fisica, Politecnico di Milano, Piazza Leonardo da Vinci 32, 20133 Milano, Italy
[6] Drug Delivery, Disposition and Dynamics, Monash Institute of Pharmaceutical Sciences, Parkville 3052, VIC, Australia
[7] Department of Pharmacy, University of Copenhagen, Universitetsparken 2, 2100 Copenhagen, Denmark

Email to: * morteza@spectophotonics.com **giuseppe@spectophotonics.com

Supplementary Information and Figures

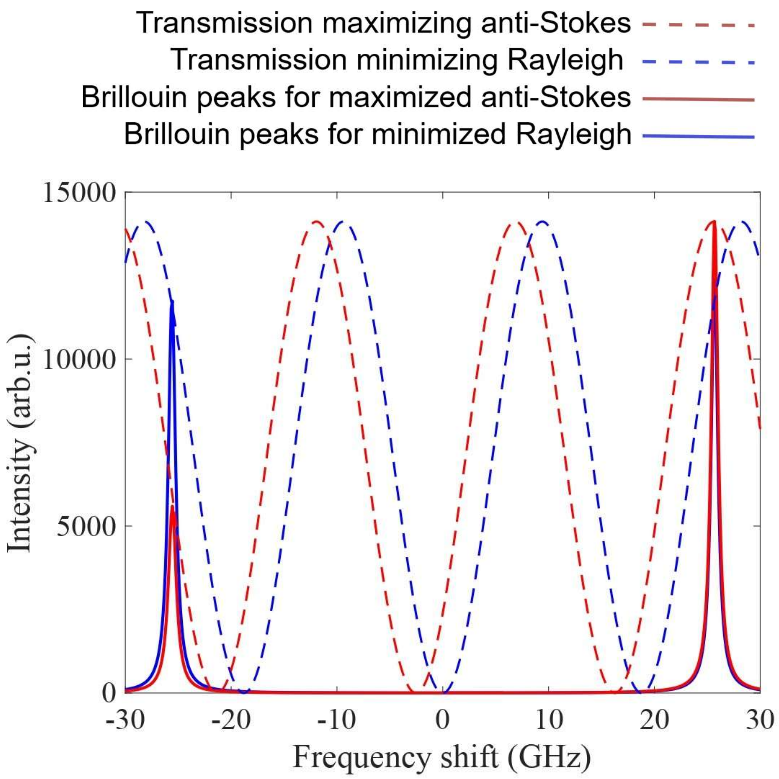

Supplementary Figure 1: Characterization of the FSR. We acquired calibrated Brillouin spectra coupling laser light into a standard optical fiber, whose spectrum featured strong Stokes and anti-Stokes Brillouin peaks from the core silica shifted by approximately 25 GHz from the central Rayleigh peak. The BIPD filter was first aligned to achieve minimum transmission at the laser (Rayleigh) frequency (dashed blue curve) and then adjusted to provide maximum transmission at the anti-Stokes Brillouin peak (dashed red curve). The reconstructed sinusoidal transfer function yielded a measured FSR of 18.9 ± 0.2 GHz (~0.63 cm^{-1}), corresponding to an estimated birefringence of $\Delta n = 0.26 \pm 0.02$. This value is in good agreement with the nominal birefringence of YVO_4 at 660 nm.

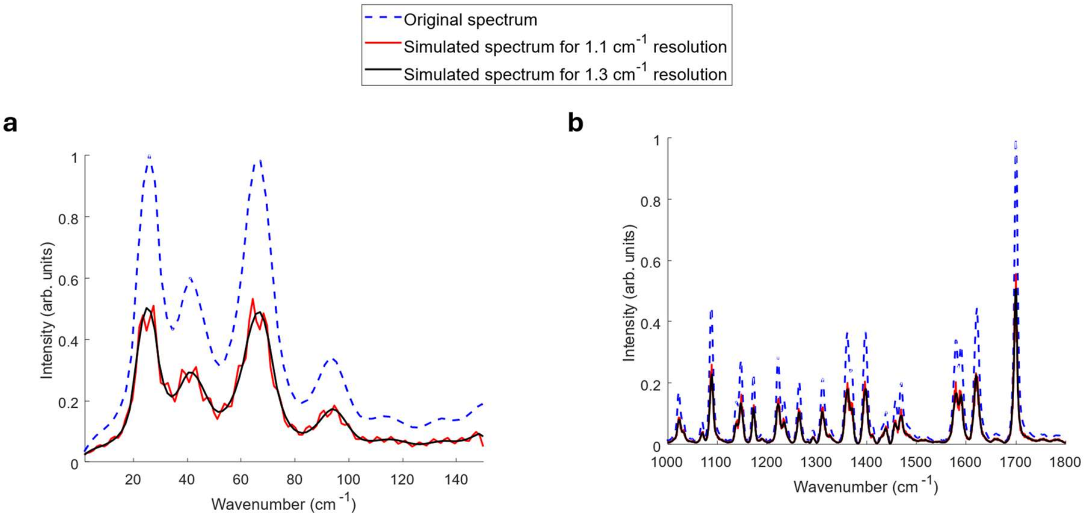

Supplementary Figure 2: Simulation of the modulation effect of the BIPD filter on (a) ULFR and (b) mid-frequency Raman spectra. A reference spectrum, measured using a commercial spectrometer, was interpolated to achieve a high number of data points (1,000,000 points; blue plot) and then multiplied by the BIPD filter's modulation function. The resulting modulated signal was integrated over intervals corresponding to the spectral resolution of the spectrometer (1.1 and 1.3 cm^{-1} in this case). Given the measured free spectral range (FSR) of 18.9 ± 0.5 GHz (~0.63 cm^{-1}) for the BIPD, a spectral resolution of ~33 GHz (1.1 cm^{-1}; less than twice the FSR) leads to a distortion in the spectral shape (red plot). In contrast, a resolution of ~39 GHz (1.3cm^{-1}; greater than twice the FSR) preserves the spectral information, resulting only in a ~50% reduction in signal intensity without introducing additional modulation effects (dark plot).

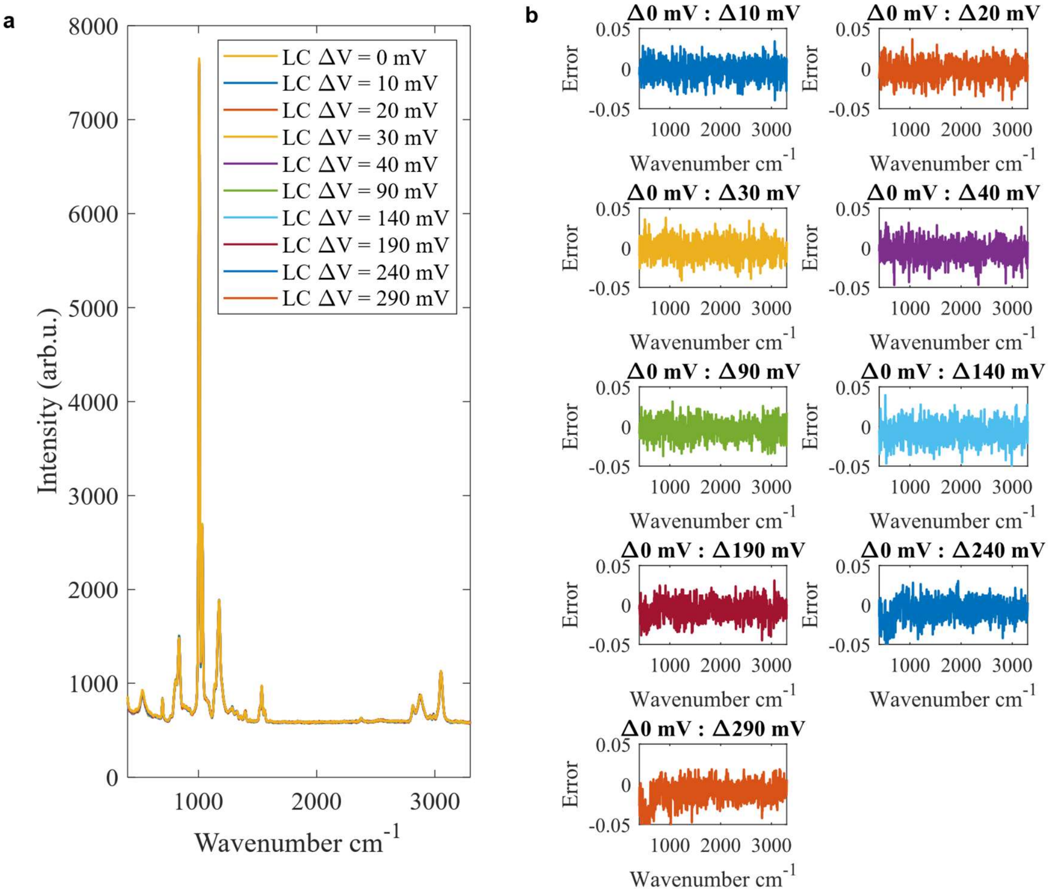

Supplementary Figure 3: **a** Raman spectra of polystyrene were acquired while translating the transfer function of the BIPD along the frequency axis. This was achieved by varying the LC voltage with different step sizes. **b** Relative error of each acquired spectrum with respect to the reference ΔV = 0 mV spectrum was calculated and plotted. The acquired spectra overlap, demonstrating that modulation of the BIPD transfer function does not result in information loss.

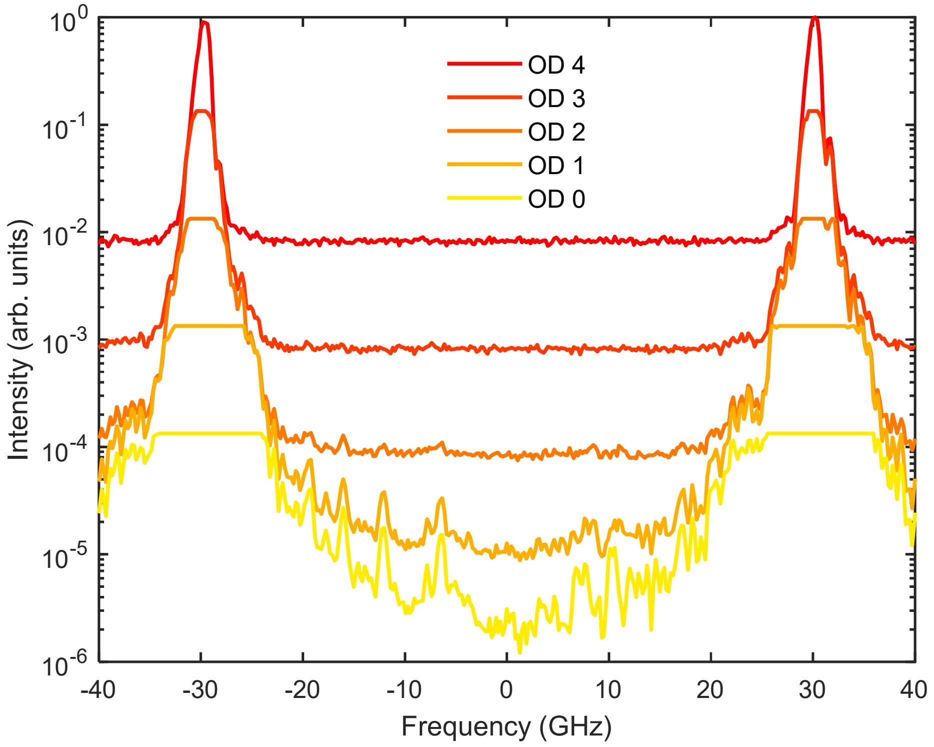

Supplementary Figure 4: Spectral contrast of our single-stage VIPA spectrometer. The VIPA was illuminated with monochromatic light at different attenuation levels using calibrated neutral density (ND) filters to overcome the limited dynamic range of the CCD camera. The resulting transmitted profiles were measured at equal exposure time. Measured spectral contrast was ~55 dB, in agreement with the theoretical expectations. Fitting the non-saturated elastic peaks with a Lorentzian function, we measured a spectral resolution of FWHM ≊ 991 MHz (0.033 cm^{-1}).

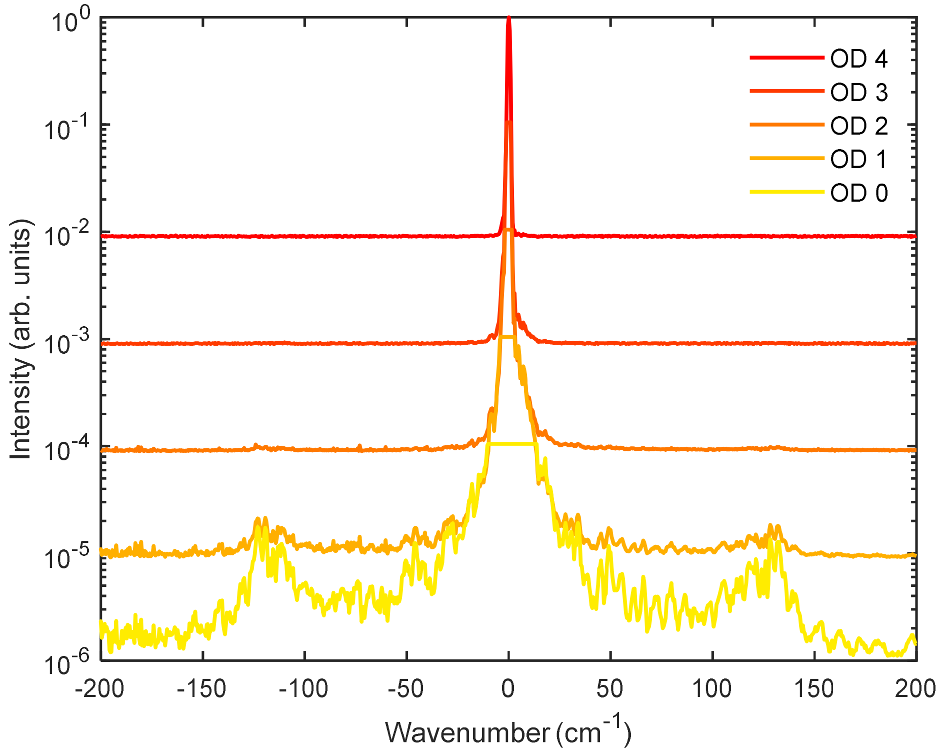

Supplementary Figure 5: Spectral contrast of our commercial grating (2400 g/mm) spectrometer. The grating was illuminated with monochromatic light at different attenuation levels using calibrated neutral density (ND) filters to overcome the limited dynamic range of the CCD camera. The resulting transmitted profiles were measured at equal exposure time. The side peaks appearing at higher intensities (OD 1 and OD 0) might be due to the stray light and reflections inside the spectrometer. Measured spectral contrast was ~50 dB. Fitting the non-saturated elastic peaks with a Lorentzian function, we measured a spectral resolution of FWHM ≅ 38.67 GHz (1.3 cm^{-1}).

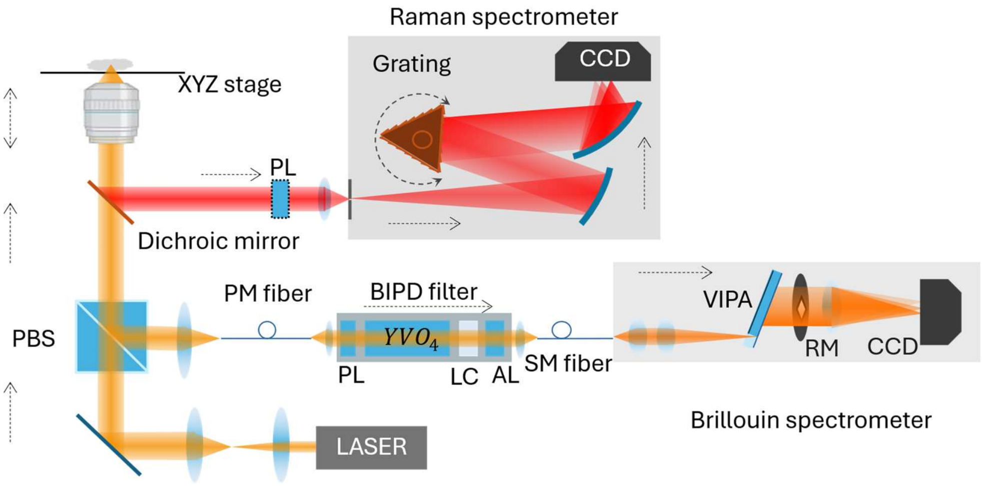

Supplementary Figure 6: Schematic of an additional optical arm built to enable collection of the Raman-scattered light without passing through the PBS and BIPD filter. In this configuration, the quarter-wave-plate was removed after the PBS to ensure a linear polarization at the illumination, and a dichroic beamsplitter was used to separate the Raman signal from the elastic background light to preserve its polarization state. PL: Polarizer, LC: Liquid Crystal, VIPA: Virtually Imaged Phased Array, RM: Rhomboidal Mask, PM: Polarization Maintaining, SM: Single Mode, AL: Analyzer.

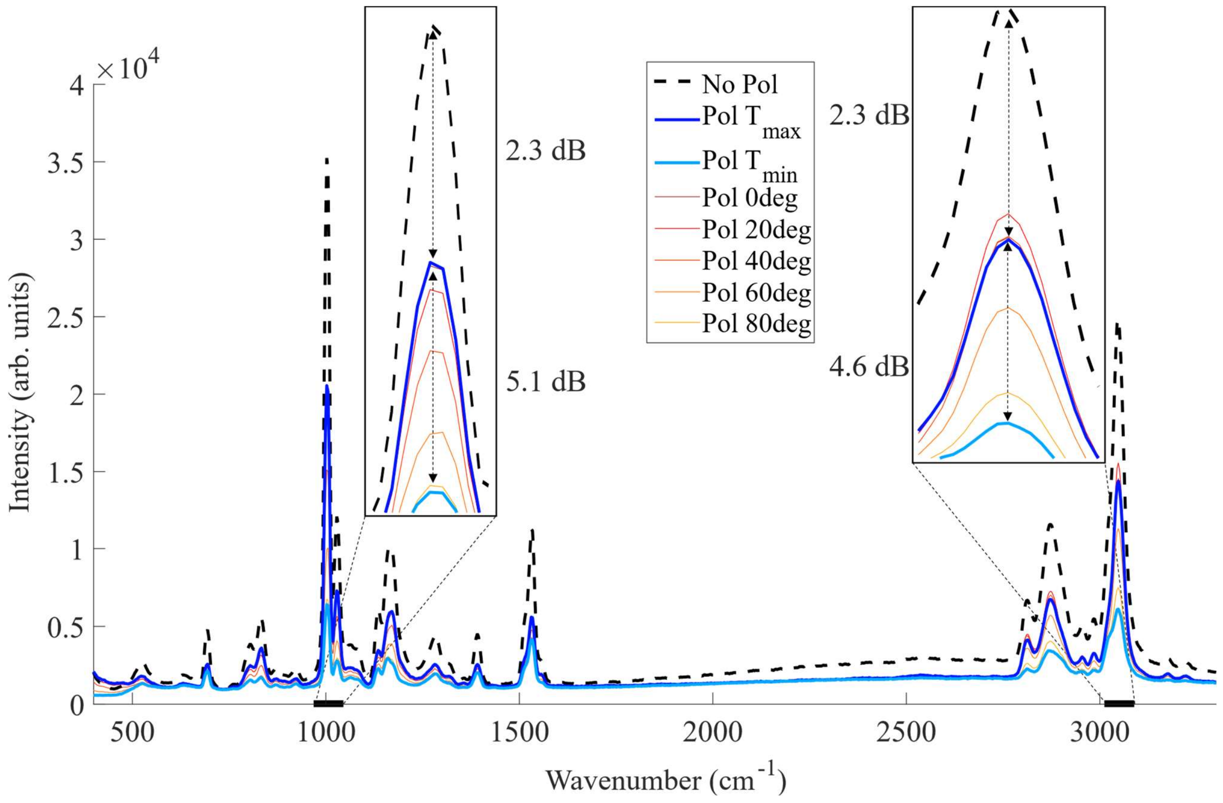

Supplementary Figure 7: Polarization analysis of the Raman-scattered light. Raman spectra of polystyrene were acquired by illuminating the sample with linear polarization, both with and without (No Pol) an analyzer whose transmission axis was rotated at different angles, including configurations that maximized (Pol T_{max}) and minimized (Pol T_{min}) the ~1000 cm^{-1} peak intensity. A 2.3 dB loss indicates that this vibrational mode is partially depolarized or not purely linearly polarized. An additional 5.1 dB loss was obtained by minimizing the transmitted signal at this peak, corresponding to a total maximum loss of 7.4 dB for this vibrational mode due to polarization selection. Furthermore, the spectra show that vibrational modes at ~1000 cm^{-1} (fingerprint region) and ~3000 cm^{-1} (C–H stretching region) exhibit distinct polarization states, as evidenced by their varying relative intensities (e.g. the red line is higher than the blue line at ~3000 cm^{-1}). The colored traces in the insets display different intensity orderings, indicating mode-dependent polarization behavior.

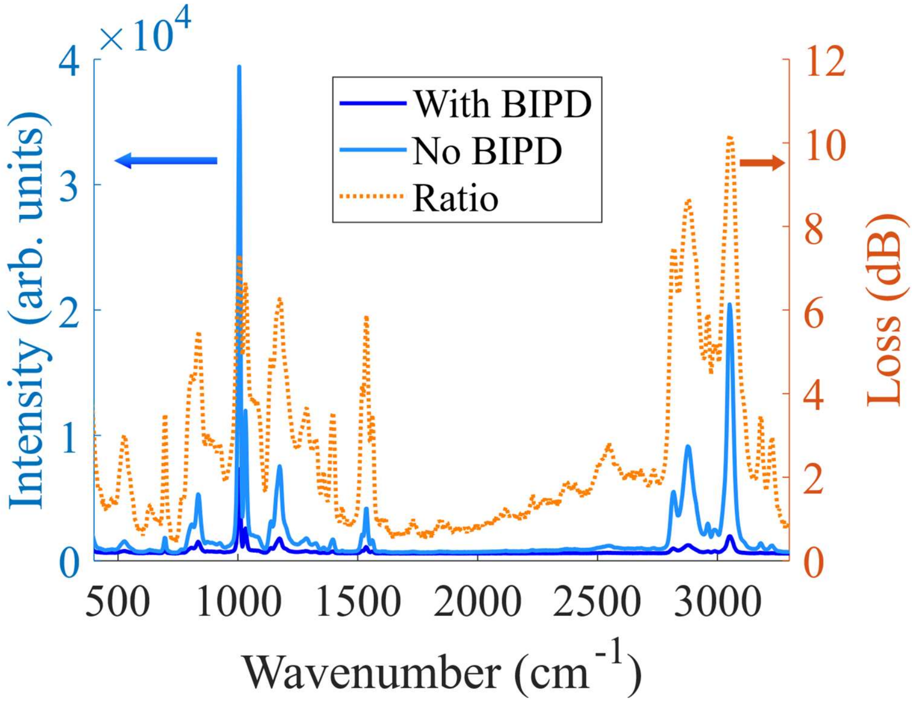

Supplementary Figure 8: Raman insertion loss introduced by the BIPD filter. Raman spectra of polystyrene were acquired in two configurations: with the signal passing through the BIPD filter and with the BIPD filter bypassed, using only a conventional edge-pass filter to suppress Rayleigh scattering. The ratio of the two spectra was calculated and is shown as a dashed line. The maximum measured loss in the fingerprint region was 7.3 dB. Considering the measured 4 dB insertion loss of the BIPD filter due to birefringent crystal absorption and fiber-coupling stages, this is very close to the expected nominal 3 dB loss arising from the intrinsic high-frequency modulation of the BIPD transfer function. The fiber-coupling loss accounts for the higher total loss observed in the fingerprint region and for the further increase to 10.2 dB in the C–H stretching region.

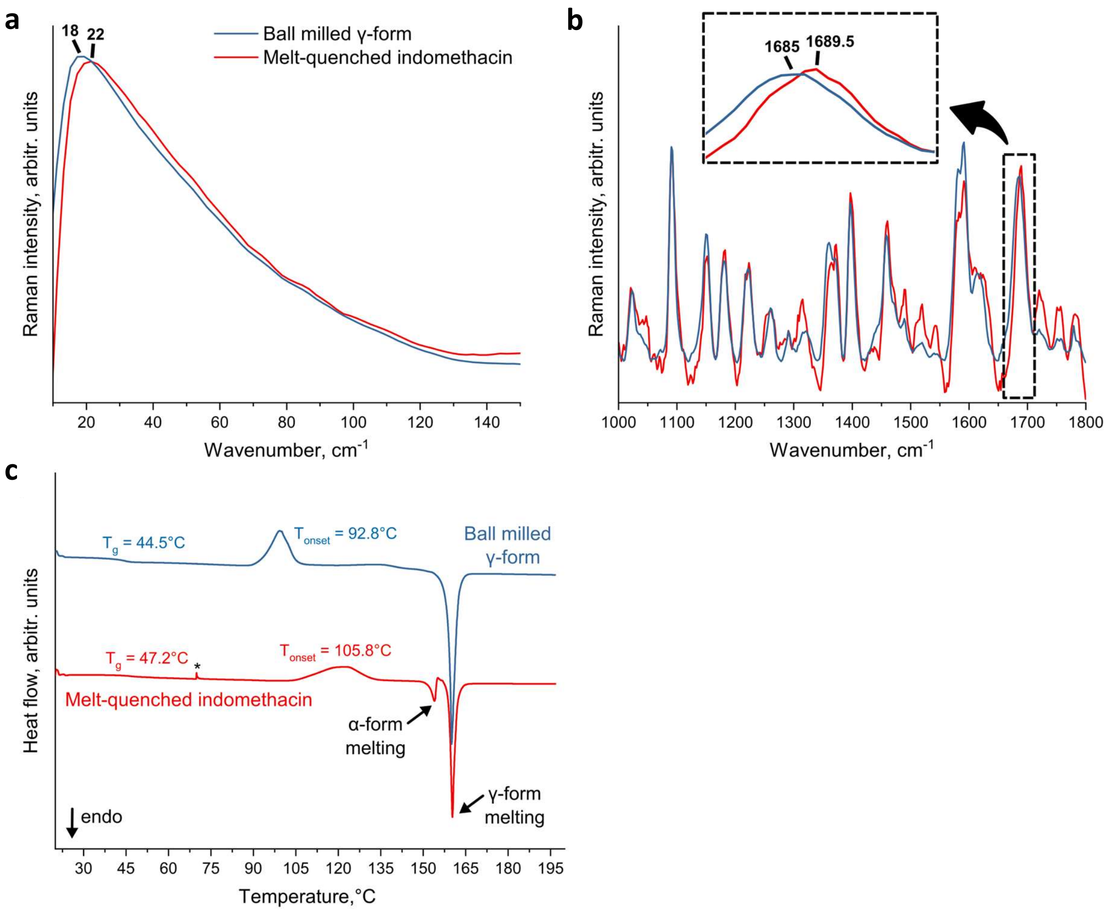

Supplementary Figure 9: Normalized (SNV corrected) (**a**) ULFR and (**b**) mid-frequency spectra examples and (**c**) DSC curves of melt-quenched indomethacin and ball milled γ-form samples. Asterisk (*) in the DSC data denotes presumed artifacts from the thermal measurement.

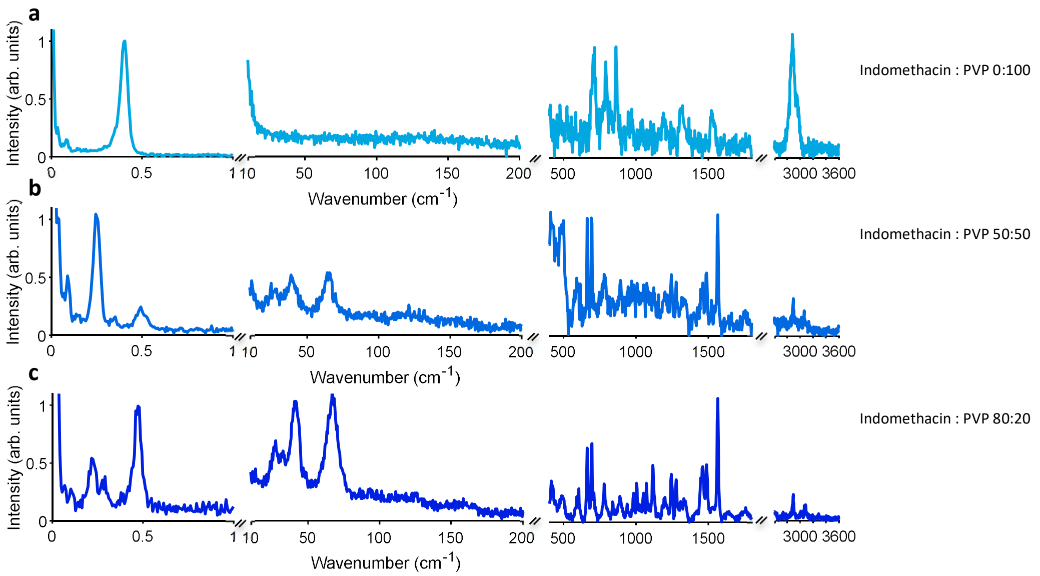

Supplementary Figure 10: Representative full spectral analysis of indomethacin-PVP mixtures acquired with full vibrational system. These data demonstrate the coherence of ULFR/Raman spectrum with the exploratory data acquired for characterization of the mixtures.

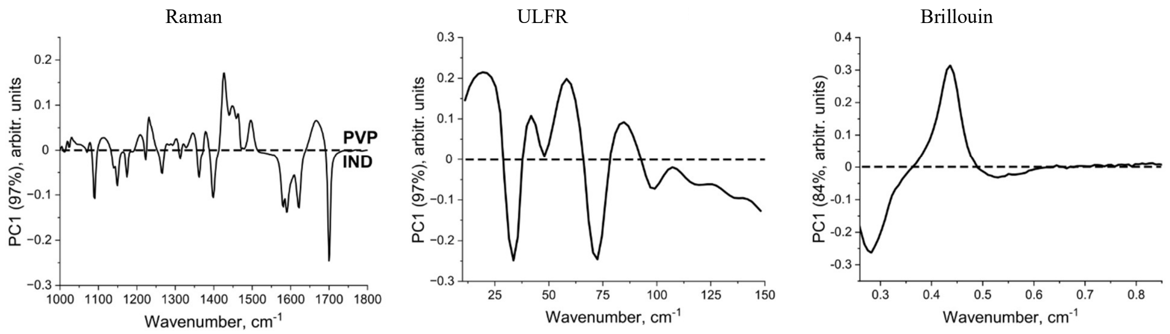

Supplementary Figure 11: Indomethacin-PVP mixture analysis. PC1 loading plots of Raman, ULFR and Brillouin spectra, respectively. Raman and ULFR spectra were acquired using dedicated ULFR / Raman instrumentation, while Brillouin spectra were acquired using full vibrational system.

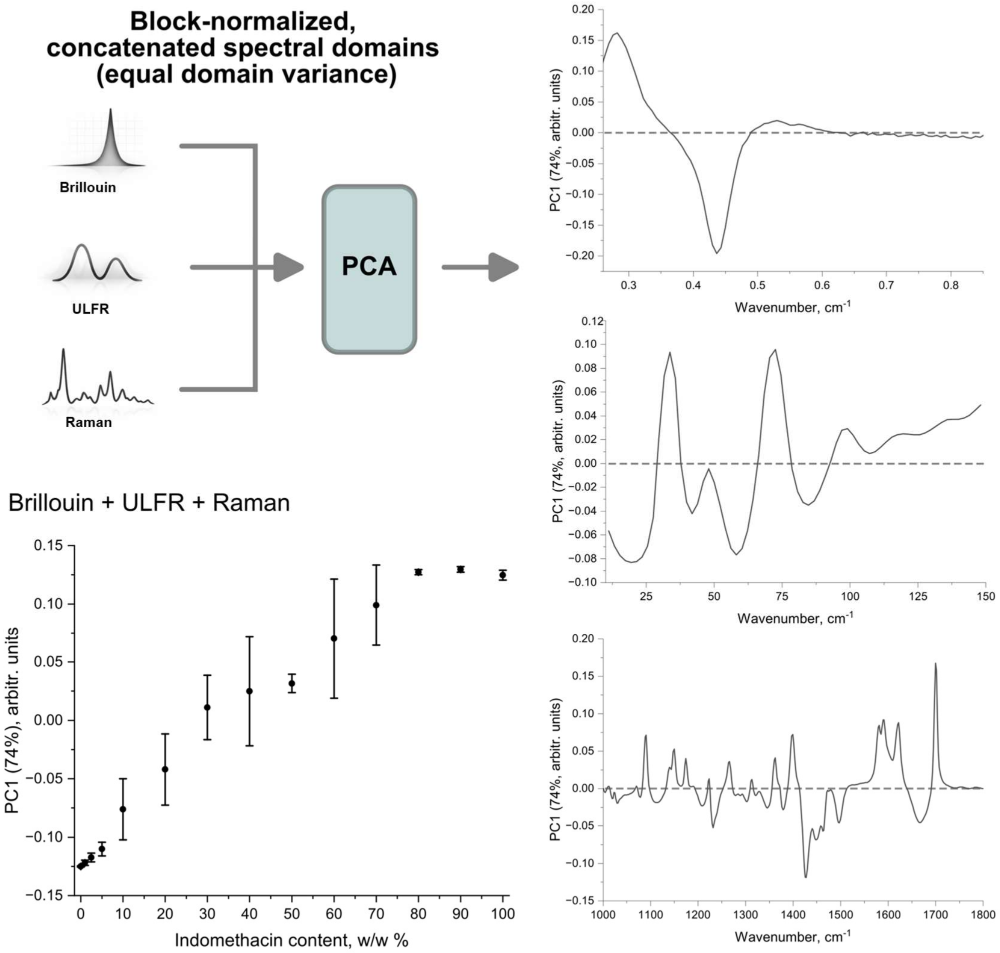

Supplementary Figure 12: PCA performed on block-normalized, concatenated spectral domains (Brillouin + ULFR + Raman) with equal variance contribution from each domain prior to fusion. The left panel shows the PC1 scores as a function of indomethacin content (w/w %), illustrating the concentration-dependent trend across the full mixture range. The right panels display the corresponding PC1 loadings for the Brillouin (top), ULFR (middle), and Raman (bottom) spectral regions, highlighting the relative contribution of each spectral domain to the first principal component.

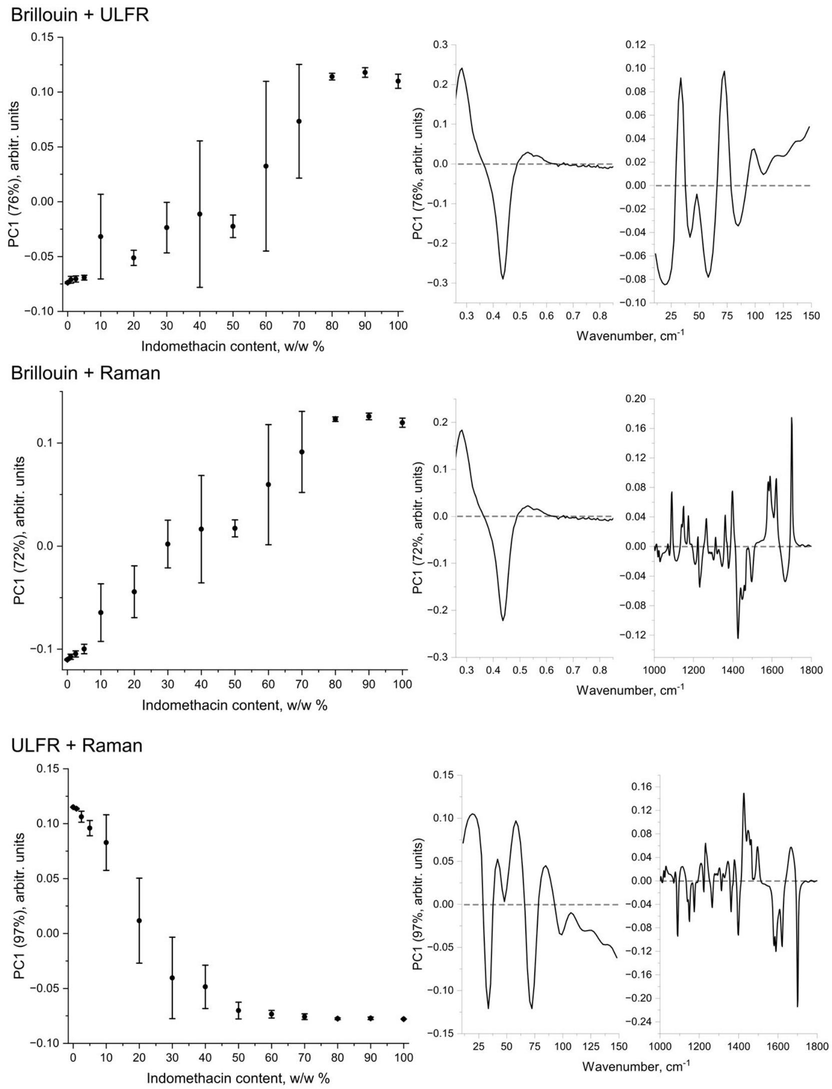

Supplementary Figure 13: PCA performed on block-normalized, concatenated spectral domains in pairwise combinations (Brillouin + ULFR, Brillouin + Raman, and ULFR + Raman), with equal variance contribution from each domain prior to fusion. In each row, the left panel shows PC1 scores as a function of indomethacin content (w/w %), while the right panels display the corresponding PC1 loadings for the respective spectral regions.

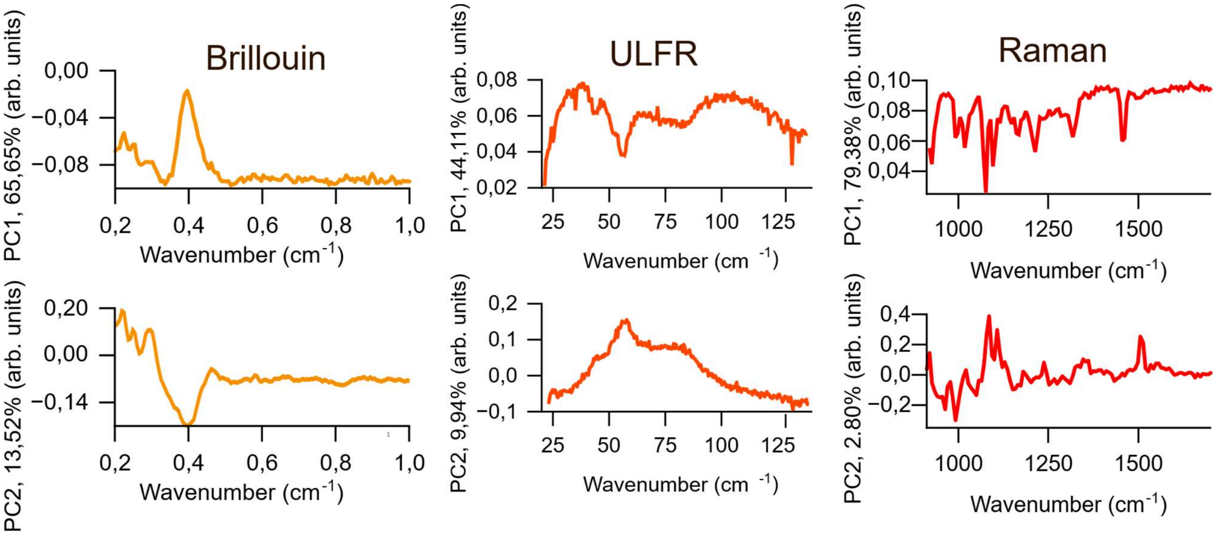

Supplementary Figure 14: Loading plots of PC1 and PC2 for Brillouin, ULFR, and Raman spectral regions along with their percentages are reported. The PCA maps in Figure 6 are plotted using PC1 of Brillouin and PC2 of ULFR and Raman spectrum and the reason for this choice is the presence of real spectral features related to Ibuprofen in these PCs (as shown in Figure 6). PC2 in Brillouin region is more representative of a pixel which lacks a peak related to Ibuprofen thus indicating the absence of Ibuprofen, furthermore a higher spectral component at lower frequencies is related to the starch content. PC1 in ULFR, is demonstrating a representative spectrum which lacks the features of a real ULFR spectrum of Ibuprofen and high intensity at lower frequencies is related to the broad vibrational density of states (VDOS) feature which is attributed to starch. PC1 in Raman region represents features that are related to the slightly shifted peaks of starch and ibuprofen coming closer to each other thus making PC1 more representative of the starch content.
