# Supplementary material for: Full vibrational spectroscopy for simultaneous mechanical, structural and chemical analysis
Source: Nat Commun. 2026 Jul 7;17:5632. doi: 10.1038/s41467-026-74558-z (PMC13342256; doi:10.1038/s41467-026-74558-z)
Supplement: Supplementary file 2 — Transparent Peer Review file [file 41467_2026_74558_MOESM2_ESM.pdf]

nature portfolio

Peer Review File

Full vibrational spectroscopy for simultaneous mechanical, structural and chemical analysis

Corresponding Author: Dr Giuseppe Antonacci

This file contains all reviewer reports in order by version, followed by all author rebuttals in order by version.

Version 0:

Reviewer comments:

Reviewer #1

(Remarks to the Author)
In this manuscript, the authors report on the integration of three spectroscopy methods to obtain optical spectra spanning 0.1 cm-1 to 4000 cm-1 to allow Brillouin, ultra-low Raman, and conventional Raman spectroscopy. This allows probing of a sample's mechanical properties, intermolecular structure, and functional group characterization. This is achieved by the development of a Birefringence-Induced Phase Delay (BIPD) filter. This was applied to characterization of pharmaceutical ingredients.

This method allows all-optical chemical and elastic characterization of sample surfaces and would find utility as a nondestructive, versatile method for material analysis. In particular, the authors demonstrated its utility to assess viscoelastic properties of indomethacin, of relevance for drug solubility and bioavailability.

I have just one minor comment: I don't see VIPA defined anywhere. Please include this.

Reviewer #3

(Remarks to the Author)
The manuscript "Full vibrational spectroscopy for simultaneous mechanical, structural, and chemical analysis" by Behrouzitabar et al. advances a technical improvement (a birefringence-induced phase delay filter) which successfully suppresses elastic scattering, a common issue in Brillouin spectroscopy, while merely reducing the signal intensity uniformly for ultra-low-frequency Raman (ULFR) and spontaneous Raman spectra. This is an interesting technical advance which enables a wide spectral imaging range encompassing the Brillouin regime (~1-10 GHz), ULFR (1-100 cm-1), and the full Raman regions up to >3,500 cm-1. The primary advantage of this method is in distinguishing ULFR, Brillouin, and elastic scattering, since Raman and Brillouin systems have been combined since at least 2015. That said, this is an interesting technical advance which can enable multi-spectral-regime characterization of diverse samples.
I have the following comments, split into major and minor concerns. If sufficiently addressed, this manuscript can merit publication in Nature Communications with significant revisions.

Major Concerns

The method suggests a 3dB reduction (half power) in Raman intensity, however, the Raman signal is passed through a polarizer (Fig 2B) which appears to significantly restrict the number of Raman photons which can enter the device, assuming the Raman photons are randomly polarized as drawn in the figure. Is this distribution of polarization as drawn (red arrows) accurate, and if so, can the authors report a more realistic number to account for this loss?

The reduction in efficiency of Raman regions is purported to be 3dB, but this is based on simulation only (Supp Figure 2). Can the authors measure the Raman intensity, both with and without the BIPD, and report the real loss? If this is not possible in the ULFR regime due to elastic scattering, it should at least be reported in the Raman regime. This is important to characterize the tradeoffs of the system, and is currently presented in simulation only. I am concerned the 3 dB claim is not sufficiently supported by data.

The method assumes that the elastic scattering from the sample will be primarily in the Rayleigh regime, however, biological applications (which may be an interest for this technology) often produce Mie scattering, which can have orthogonal components to its polarization, even in pure backscattering. How will this affect the measurement?

Given that this method relies on the polarization of Rayleigh, Brillouin, and Raman scattered photons (all light passes through a polarizer before the birefringent prism) there needs to be better description and characterization of the degree of polarization of these scattered components and their rejection (or lack thereof) by the polarizer and by the birefringent prism. This should include both a theoretical basis (which of these components will have which expected polarization) and empirical measurements.

Are the technical samples reported here of sufficient medical or scientific relevance? It seems that the samples were chosen due to their spectral signatures in the 3 wavelength regions, but the manuscript would have stronger impact if a challenging problem (i.e. detecting small % changes in concentration of drug, rather than distinguishing different mixtures of very different drugs) was demonstrated. I am not convinced (as a reader) that pharmaceutical manufacturers have trouble telling their drugs apart.

Figure 5 – The principle component analysis is interesting, but I am not sure how compelling the results are. It seems the ability to distinguish different substances is most pronounced in the Raman region, and ULFR and Brillouin perform slightly worse. Is it possible to conduct a PCA analysis with *only Raman* versus *all three spectra combined*, and compare the two, to see how much utility is added by measuring all three regions at once? This may provide strong justification for the utility of the system.

Figure 6 – The PCA maps appear largely similar to each other. It isn't clear to me how adding additional spectral regions is adding to the analysis of this sample. The significance of this measurement (combining all 3 regions) should be better justified, and if not significant, a better sample should be chosen.

Supp Figure 2 – The caption says "detected" but this is simulated data. Also, it would be useful to divide the resulting spectrum by the original to assess the consistency to the 3 dB reduction. I recommend doing this with real (rather than simulated) data as mentioned above, though the simulated data is fine to include to demonstrate the principle.

Minor Concerns.
Page 3 – name the variable v_B for those unfamiliar.
Page 6 – "these filters lack the bandwidth to isolate Brillouin/ULFR from elastic background". The purpose of these filters is to isolate Brillouin from Raman, and removal of the elastic peak is generally accomplished in some other way. This phrasing is a bit confusing to me and seems to conflate two issues.
Page 5 – "…these spectral regimes may offer more valuable insights to enhance the precision…" This sentence is a bit vague.
Page 6, Reference 32. There were earlier implementations of combined Raman and Brillouin, e.g. DOI: 10.1021/acs.analchem.5b02104
Alternative approaches to mechanical/structural imaging should be cited, e.g. https://doi.org/10.1038/s41598-022-23049-4
Page 6 – "lockin" -> "locking
The inset in Figure 2C is helpful. A similar inset showing modulation in the ULFR regime would be useful.
Fig 2 – Rayliegh *photons are* polarized
Page 9. The measured spectral resolution is 1 GHz. Is this sufficient for most Brillouin applications? Can this be improved in this study by deconvolving with the instrument response function, as is commonly done in Brillouin studies?
Page 10. "Convert" – a better word might be "calibrate"
Page 14 – Ball-milled, crystalline, and MQ should be introduced and better explained – as a reader it is not clear why these distinctions are important.
Page 18 – Recommend introducing the term "endmembers" in greater detail.
Figure 6(b,c) – this is showing PC2 – should this be PC1?
Page 21 – "Our finding show…"
A discussion of the relevance of this technology to applications in biology would be welcome.
Supp Figure 1 – A legend explaining line colors would be helpful to aid the reader.
Supp Figure 2 – The legend and caption have different numbers for resolution.
Supp Figure 5(a,b). These lines closely overlap. Are there error bars on these measurements? If not, it is difficult to claim a clear distinction between the two.

Version 1:

Reviewer comments:

Reviewer #1

(Remarks to the Author)
The authors have addressed my comments and the revised manuscript is suitable for publication.

Reviewer #3

(Remarks to the Author)

The manuscript has been substantially improved as a result of these changes; I recommend this manuscript for publication.

Open Access This Peer Review File is licensed under a Creative Commons Attribution 4.0 International License, which permits use, sharing, adaptation, distribution and reproduction in any medium or format, as long as you give appropriate credit to the original author(s) and the source, provide a link to the Creative Commons license, and indicate if changes were made.
In cases where reviewers are anonymous, credit should be given to 'Anonymous Referee' and the source.
The images or other third party material in this Peer Review File are included in the article’s Creative Commons license, unless indicated otherwise in a credit line to the material. If material is not included in the article’s Creative Commons license and your intended use is not permitted by statutory regulation or exceeds the permitted use, you will need to obtain permission directly from the copyright holder.
To view a copy of this license, visit https://creativecommons.org/licenses/by/4.0/

Response to Reviewers' Comments

Reviewer #1 (Remarks to the Author):

In this manuscript, the authors report on the integration of three spectroscopy methods to obtain optical spectra spanning 0.1 cm^{-1} to 4000 cm^{-1} to allow Brillouin, ultra-low Raman, and conventional Raman spectroscopy. This allows probing of a sample's mechanical properties, intermolecular structure, and functional group characterization. This is achieved by the development of a Birefringence-Induced Phase Delay (BIPD) filter. This was applied to characterization of pharmaceutical ingredients.
This method allows all-optical chemical and elastic characterization of sample surfaces and would find utility as a nondestructive, versatile method for material analysis. In particular, the authors demonstrated its utility to assess viscoelastic properties of indomethacin, of relevance for drug solubility and bioavailability.

We thank the reviewer for the positive evaluation of our manuscript and for recognizing the potential of integrating Brillouin, ultra-low-frequency Raman, and conventional Raman spectroscopy within a single platform to provide complementary mechanical, structural, and chemical information. We hope that the revisions in the manuscript have further clarified the methodology and addressed the reviewer's comments.

I have just one minor comment: I don't see VIPA defined anywhere. Please include this.

We thank the reviewer for noticing this.

> **Actions taken** – We have added the following definition of VIPA on page 6 of the Introduction section: "*Virtually Imaged Phased Array (VIPA)-based*".

Reviewer #3 (Remarks to the Author):

The manuscript "Full vibrational spectroscopy for simultaneous mechanical, structural, and chemical analysis" by Behrouzitabar et al. advances a technical improvement (a birefringence-induced phase delay filter) which successfully suppresses elastic scattering, a common issue in Brillouin spectroscopy, while merely reducing the signal intensity uniformly for ultra-low-frequency Raman (ULFR) and spontaneous Raman spectra. This is an interesting technical advance which enables a wide spectral imaging range encompassing the Brillouin regime (~1-10 GHz), ULFR (1-100 cm^{-1}), and the full Raman regions up to >3,500 cm^{-1}. The primary advantage of this method is in distinguishing ULFR, Brillouin, and elastic scattering, since Raman and Brillouin systems have been combined since at least 2015. That said, this is an interesting technical advance which can enable multi-spectral-regime characterization of diverse samples.
I have the following comments, split into major and minor concerns. If sufficiently addressed, this manuscript can merit publication in Nature Communications with significant revisions.

We thank the reviewer for the careful evaluation of our manuscript and for recognizing the technical advance introduced by the birefringence-induced phase delay (BIPD) filter and its potential. We appreciate the reviewer's constructive feedback and the detailed comments provided. We have carefully addressed the points raised and revised the manuscript accordingly. We hope that the revised version satisfactorily clarifies the methodology and addresses the reviewer's concerns.

Major concerns

The method suggests a 3dB reduction (half power) in Raman intensity, however, the Raman signal is passed through a polarizer (Fig 2B) which appears to significantly restrict the number of Raman photons which can enter the device, assuming the Raman photons are randomly polarized as drawn in the figure. Is this distribution of polarization as drawn (red arrows) accurate, and if so, can the authors report a more realistic number to account for this loss?

We thank the reviewer for the observation. In principle, all scattered contributions entering the filter are linearly polarized because they are first reflected by the PBS, which effectively acts as a polarizer. On the other hand, Fig. 2b provides a more qualitative representation of the polarization state of each scattered contribution, highlighting the fact that, differently from the Rayleigh and Brillouin signals, the Raman scattered light is typically randomly polarized or partially depolarized depending on the material and specific vibrational modes. The loss related to the polarization selection in our setup is thus not given by the polarizer placed at the entrance of the filter but rather by the PBS. In particular, the PBS acts as a linear polarizer and therefore introduces losses if the light being inelastically scattered is not linearly polarized along the transmission axis of the PBS, as in the case of the Raman scattered light.

> **Actions taken** – To improve the clarity of the Raman polarization state shown in Figure 2b, we have updated the figure (Figure R1b) by adding schematic representations of circular and elliptical polarization states of the Raman signal, which can occur in spontaneous Raman scattering according to both theory and our measurements. Although the reflection from the PBS ideally results in a linearly polarized state, the purpose of this illustration is to provide a qualitative overview of the possible polarization processes involved.

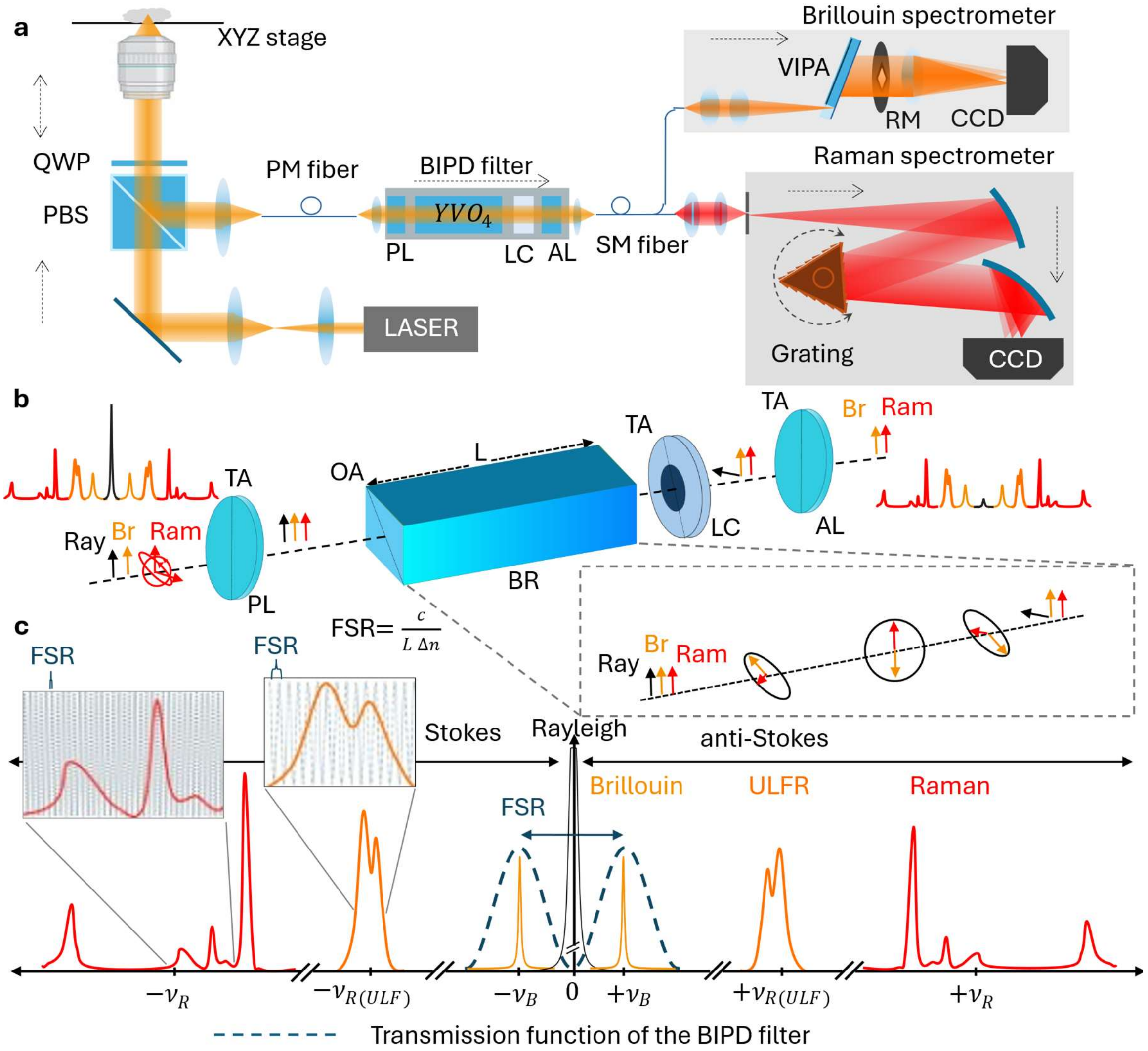

Figure R1 - Multimodal Brillouin and Raman confocal system. ***a*** *Schematic of the custom confocal multimodal Brillouin and Raman system. Backscattered elastic and inelastic signals are coupled into a single-mode polarization-maintaining (PM) fiber acting as a confocal pinhole. Light transmitted is filtered by the BIPD module (****b****). This includes a polarizer (PL) with transmission axis (TA) aligned to the input polarization and a birefringent (BR) crystal. A liquid crystal (LC) retarder tunes the phase delay to ensure that the elastic signal is polarized orthogonally to the analyzer's (AL) TA. The filter's transmission is maximized at the expected Brillouin frequency with an appropriate choice of the FSR (****c****). This causes high-frequency modulation in ULFR and Raman regions resulting in a nominal optical loss of 3 dB, but without loss in spectral information. The light transmitted by the filter is coupled to a single-mode (SM) fiber to deliver the signal to the spectrometers. Specifically, the Brillouin spectrometer consists of a single-stage VIPA etalon assisted with a rhomboidal mask (RM) for contrast enhancement, while the Raman spectrometer is a grating-based commercial unit.*

To investigate how the Raman signal changes its polarization state with respect to the illumination polarization, we built an additional optical arm (Figure R2, new Supplementary Fig. 6) that enables collection of the Raman-scattered light without passing through the PBS. In this configuration, we removed the quarter-wave-plate after the PBS and used a standard dichroic beamsplitter to preserve the polarization state of the Raman-scattered light in collection prior to polarization analysis. Such analysis reveals how the polarization state of different vibrational modes changes with respect to the linear excitation polarization, thus providing a more realistic evaluation of the losses in the system. The polarization analysis is performed by rotating a polarizer before the spectrometer.
The schematic has been added as Supplementary Fig 6.

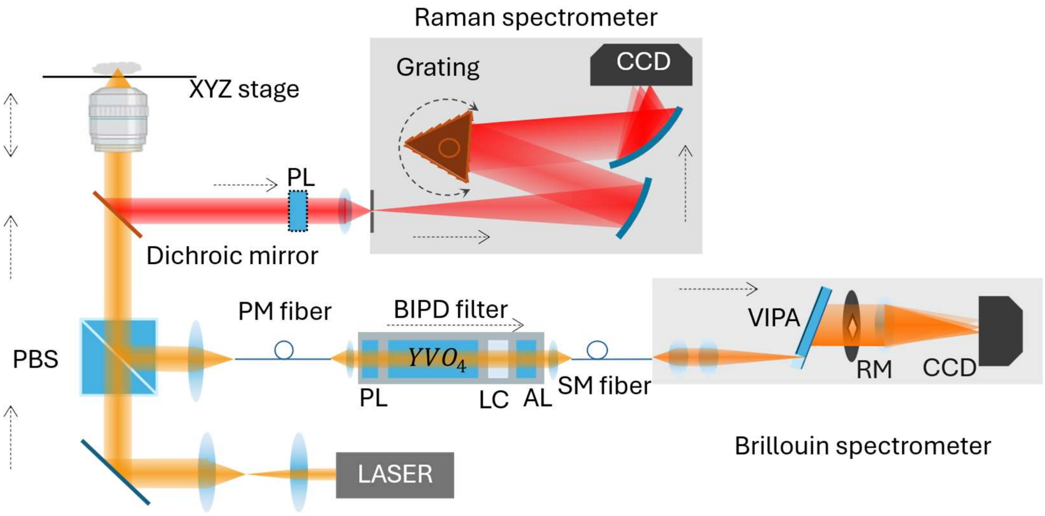

Figure R2 - Schematic of an additional optical arm built to enable collection of the Raman-scattered light without passing through the PBS and BIPD filter. In this configuration, the quarter-wave-plate was removed after the PBS to ensure a linear polarization at the illumination, and a dichroic beamsplitter was used to separate the Raman signal from the elastic background light to preserve its polarization state. PL: Polarizer, LC: Liquid Crystal, VIPA: Virtually Imaged Phased Array, RM: Rhomboidal Mask, PM: Polarization Maintaining, SM: Single Mode, AL: Analyzer.

For the polarization analysis, the Raman spectrum of bulk polystyrene was acquired with the analyzer transmission axis set at different angles (Figure R3, new Supplementary Fig. 7). Spectra were recorded without an analyzer (No Pol) and with the analyzer oriented at various angles. The spectra were further measured under conditions that maximized (Pol T_{max}) and minimized (Pol T_{min}) the intensity of the peak at ~1000 cm^{-1}. The results show that the vibrational mode corresponding to the ~1000 cm^{-1} peak exhibits a minimum of 2.3 dB loss, indicating a partially depolarized or not purely linearly polarized state. This peak was further suppressed by minimizing the transmission through the analyzer, resulting in a total loss of 7.4 dB due to polarization selection.
Moreover, different vibrational modes (e.g., ~1000 cm^{-1} in the fingerprint region and ~3000 cm^{-1} in the C–H stretching region) exhibit distinct polarization states, as reflected by their varying relative intensities at different analyzer angles. The corresponding colored traces in the insets show different intensity orderings, indicating mode-dependent polarization behavior.
The results have been added as Supplementary Fig. 7.

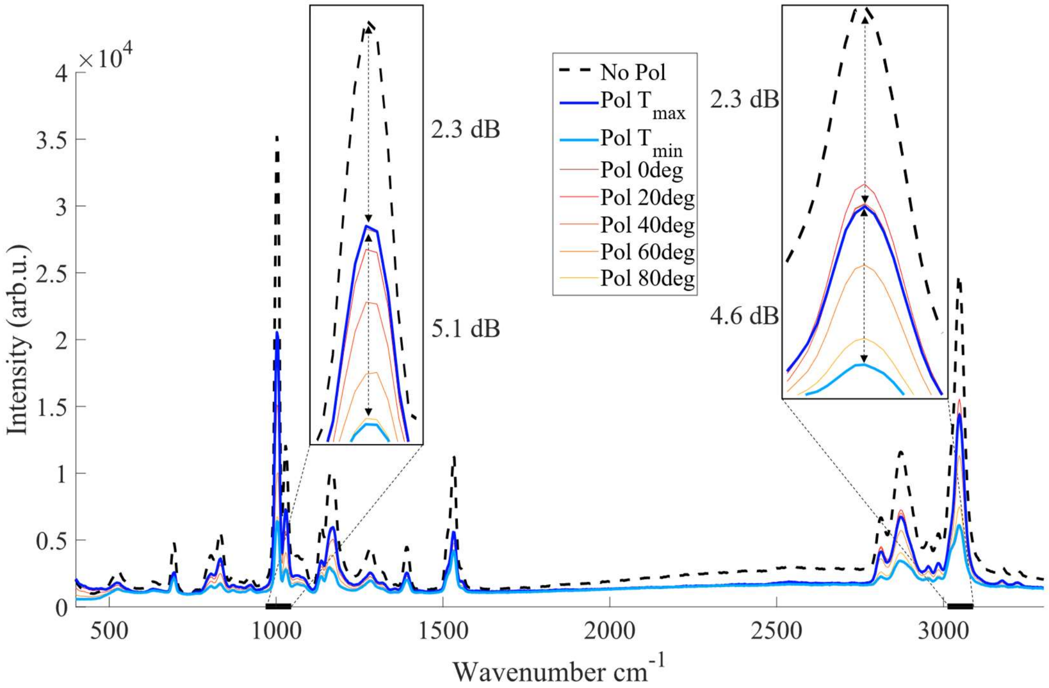

Figure R3 - Polarization analysis of the Raman-scattered light. Raman spectra of polystyrene were acquired by illuminating the sample with linear polarization, both with and without (No Pol) an analyzer whose transmission axis was rotated at different angles, including configurations that maximized (Pol T_{max}) and minimized (Pol T_{min}) the ~1000 cm^{-1} peak intensity. A 2.3 dB loss indicates that this vibrational mode is partially depolarized or not purely linearly polarized. An additional 5.1 dB loss was obtained by minimizing the transmitted signal at this peak, corresponding to a total maximum loss of 7.4 dB for this vibrational mode due to polarization selection. Furthermore, the spectra show that vibrational modes at ~1000 cm^{-1} (fingerprint region) and ~3000 cm^{-1} (C–H stretching region) exhibit distinct polarization states, as evidenced by their varying relative intensities (e.g. the red line is higher than the blue line at ~3000 cm^{-1}). The colored traces in the insets display different intensity orderings, indicating mode-dependent polarization behavior.

We have added a discussion on page 11 of the results section as follows:

"*To investigate the effect of polarization selection on Raman modes, we implemented an additional optical arm (Supplementary Fig. 6) to analyze the polarization state of the Raman vibrational modes of polystyrene using an analyzer. Results show that a loss of 2.3 – 7.4 dB is introduced due to polarization selection depending on the specific vibrational mode (Supplementary Fig. 7).*"

The reduction in efficiency of Raman regions is reported to be 3dB, but this is based on simulation only (Supp Figure 2). Can the authors measure the Raman intensity, both with and without the BIPD, and report the real loss? If this is not possible in the ULFR regime due to elastic scattering, it should at least be reported in the Raman regime. This is important to characterize the tradeoffs of the system and is currently presented in simulation only. I am concerned that the 3 dB claim is not sufficiently supported by data.

We thank the reviewer for this valuable suggestion. The reported 3 dB loss associated with high-frequency modulation of the BIPD filter at higher wavelengths is a nominal value obtained from simulations. In response to the reviewer's comment, we performed additional experiments to determine the actual experimental loss by comparing the Raman intensity with and without the BIPD filter.

Actions taken - To quantify the actual loss, we acquired the Raman spectrum of bulk polystyrene under two configurations: with the BIPD inserted in the detection path and with the BIPD bypassed

and replaced by a conventional edge-pass filter as a standard solution to remove the Rayleigh component. In the second configuration, unlike the first, no fiber was used after the edge-pass filter, thereby eliminating the need for fiber coupling. Corresponding spectra for both configurations, together with their intensity ratio expressed in dB, have been presented to determine the measured loss of 7.3 – 10.2 dB which comprises losses due to crystal absorption and fiber coupling as well as high-frequency modulation (Figure R4, new Supplementary Fig. 8).
The results have been included in Supplementary Fig. 8, and a corresponding discussion has been added on page 11 of the results section as follows:

"*To evaluate the high-frequency modulation losses predicted by simulations, we compared the Raman signal acquired with and without the BIPD filter in the detection path, finding a maximum measured loss of 7.3 dB in the fingerprint region around 1000 cm^{-1}. Considering a 4 dB insertion loss caused by the absorption of the birefringent crystal and the fiber-coupling stages, the residual loss is close to the expected 3 dB loss arising from the intrinsic high-frequency modulation, with total losses reaching a maximum value of 10.2 dB in the C–H stretching region (Supplementary Fig. 8).*"

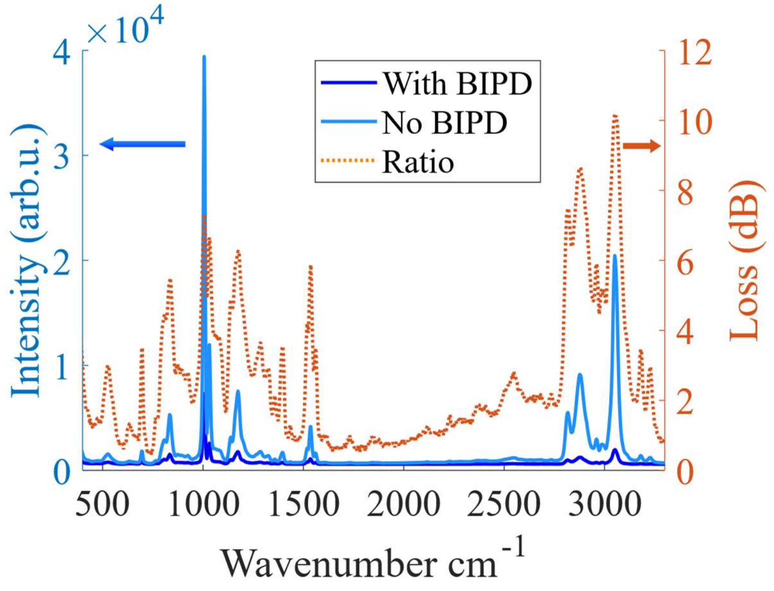

Figure R4 – Raman insertion loss introduced by the BIPD filter. Raman spectra of polystyrene were acquired in two configurations: with the signal passing through the BIPD filter and with the BIPD filter bypassed, using only a conventional edge-pass filter to suppress Rayleigh scattering. The ratio of the two spectra was calculated and is shown as a dashed line. The maximum measured loss in the fingerprint region was 7.3 dB. Considering the measured 4 dB insertion loss of the BIPD filter due to birefringent crystal absorption and fiber-coupling stages, this is very close to the expected nominal 3 dB loss arising from the intrinsic high-frequency modulation of the BIPD transfer function. The fiber-coupling loss accounts for the higher total loss observed in the fingerprint region and for the further increase to 10.2 dB in the C–H stretching region.

To further support the results of simulation which indicates that the BIPD does not cause information loss, we shifted the BIPD transfer function by varying the LC voltage to verify that the filter does not suppress Raman modes in the high-frequency region (Figure R5, new Supplementary Fig. 3). Raman spectra of polystyrene were acquired while translating the BIPD transfer function along the frequency axis using different LC voltage step sizes. The resulting spectra overlap, demonstrating that modulation of the BIPD transfer function does not lead to loss of Raman information.
We have added the results in Supplementary Fig. 3 and a description on page 10 of the results section as follows:

"*To further support this, we shifted the BIPD transfer function by varying the LC voltage to verify that its high-frequency modulation response does not suppress Raman modes (Supplementary Fig. 3).*"

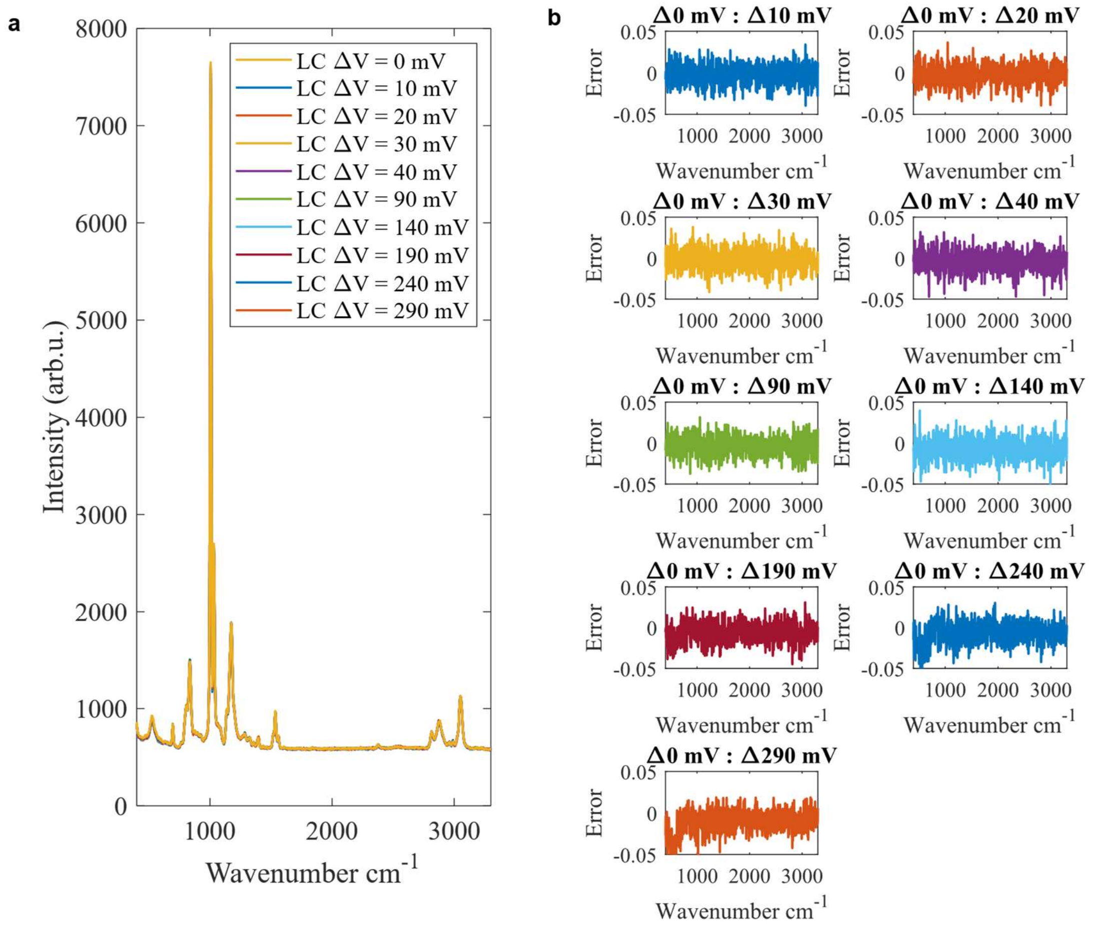

*Figure R5 – **a** Raman spectra of polystyrene were acquired while translating the transfer function of the BIPD along the frequency axis. This was achieved by varying the LC voltage with different step sizes. **b** Relative error of each acquired spectrum with respect to the reference ΔV = 0 mV spectrum was calculated and plotted. The acquired spectra overlap, demonstrating that modulation of the BIPD transfer function does not result in information loss.*

The method assumes that the elastic scattering from the sample will be primarily in the Rayleigh regime, however, biological applications (which may be an interest for this technology) often produce Mie scattering, which can have orthogonal components to its polarization, even in pure backscattering. How will this affect the measurement?

We thank the reviewer for pointing out this important aspect, which allows us to provide further clarification on the working principle of the filter for the reader.
Rayleigh scattering occurs when light interacts with particles that are significantly smaller than the wavelength of the incident radiation and typically results in a highly polarized scattered field. In contrast, Mie scattering arises from particles whose dimensions are comparable to or larger than the wavelength, and it can introduce additional polarization components depending on the scattering geometry and particle properties.
In our system, the polarizer placed at the input of the BIPD filter enforces a well-defined linear polarization state for all field components entering the device. Specifically, it suppresses orthogonal polarization components, thereby ensuring homogeneous linear polarization at the filter input. Consequently, irrespective of the initial polarization state of the background light, whether originating from Rayleigh scattering, Mie scattering, or Fresnel reflections, the field is projected onto a single linear polarization state before interacting with the filter's components. Therefore, the operational performance and the functionality of the filter remain unaffected by the polarization characteristics of the incident background light.

> **Actions taken** - We have added the following text on page 7 of the introduction section:
>
> "*In particular, the BIPD effectively rejects background signals regardless of their polarization state or physical origin. Independent of whether the background arises from Rayleigh or Mie scattering or from Fresnel reflections, the polarizer at the input of the BIPD imposes a linear polarization state on the incident light, thereby preserving the operating principle of the filter.*"

Given that this method relies on the polarization of Rayleigh, Brillouin, and Raman scattered photons (all light passes through a polarizer before the birefringent prism) there needs to be better description and characterization of the degree of polarization of these scattered components and their rejection (or lack thereof) by the polarizer and by the birefringent prism. This should include both a theoretical basis (which of these components will have which expected polarization) and empirical measurements.

We thank the reviewer for highlighting the importance of a more detailed characterization of the polarization of the scattered components. The polarizer at the input of the BIPD filter ensures a well-defined linear polarization for all field components entering the filter, and consequently, the working principle of the BIPD filter is not affected by the intrinsic polarization properties of the scattered light. Nevertheless, characterization of the polarization of the scattered light is important to assess potential losses introduced by polarization selection, which occurs primarily through reflection at the PBS and subsequently at the input polarizer of the BIPD filter.

> **Actions taken** - To provide additional clarity on both the theoretical basis and the experimental characterization of the polarization states of the different scattering processes, we discussed the underlying theoretical mechanisms and performed additional measurements to quantify the polarization state of the Raman scattered light, addressing the reviewer's comments above.
> We have added the following text on pages 10 and 11 of the results section.
>
> "*In this system, additional optical losses for the Raman signal arise because the polarization of scattered light depends on both the scattering mechanism and the excitation–detection geometry, leading to the rejection of certain components by the PBS before the input polarizer of the BIPD filter. For Rayleigh scattering, the polarization is largely preserved in isotropic scatterers, as it reflects the induced dipole moment. In Brillouin scattering, the polarization depends on the type of phonon involved, with longitudinal acoustic phonons, common in 180° backscattering geometries, typically maintaining the incident polarization. Raman scattering exhibits more complex behavior, as the polarization of the scattered light is determined by the symmetry of the vibrational modes.*

Totally symmetric modes tend to preserve polarization, whereas non-totally symmetric modes show partial depolarization[50-52]. Because the polarization of scattered light depends on both sample properties and the measurement configuration, polarization selection can contribute to additional losses."

We conducted experiments to support the statements discussed and added the results to the reviewer's remarks above.

We have added new references to the Reference section as follows:

50. Peter, Y. & Cardona, M. Fundamentals of semiconductors: physics and materials properties (Springer Science & Business Media, 2010).
51. Tuschel, D. Practical group theory and raman spectroscopy, part i: Normal vibrational modes. Spectroscopy 29, 14 (2014).
52. Dennis, M. R. A three-dimensional degree of polarization based on rayleigh scattering. Journal of the Optical Society of America A 24, 2065–2069 (2007).

Are the technical samples reported here of sufficient medical or scientific relevance? It seems that the samples were chosen due to their spectral signatures in the 3 wavelength regions, but the manuscript would have stronger impact if a challenging problem (i.e. detecting small % changes in concentration of drug, rather than distinguishing different mixtures of very different drugs) was demonstrated. I am not convinced (as a reader) that pharmaceutical manufacturers have trouble telling their drugs apart.

We thank the reviewer for this insightful comment and apologize for any confusion caused by the presentation of the data. While Figure 4 was intended as a demonstration of the technique's ability to distinguish between different drugs, the main goal of this figure is to show that this approach can differentiate samples of the same drug prepared under different processing conditions, reflecting distinct preparation histories. Furthermore, Figure 5 highlights the technique's capability to address a more challenging and practically relevant problem: detecting small changes in concentration across an extended range, particularly at higher concentrations, which cannot be reliably achieved using only high-frequency vibrational components. These results demonstrate that the method can tackle issues of practical pharmaceutical and scientific significance beyond simple differentiation of clearly distinct compounds.

Actions taken - To provide additional clarity on the scientific relevance of the reported samples, we have added the following discussion on page 14 of the results section.

"*These are widely used and extensively studied APIs, frequently employed as model systems in solid-state and formulation research. The scenarios addressed in this work reflect significant challenges in pharmaceutical development. For example, amorphous phases often lack clear structural fingerprints, making it difficult to demonstrate meaningful differences using conventional analytical techniques, despite their impact on stability, manufacturability, and bioavailability[53]. This issue is also relevant from a regulatory and intellectual property perspective. The sensitivity of the Brillouin scattering signal to viscoelastic variations therefore provides complementary information that is difficult to access with conventional Raman-based approaches alone.*"

We have added a new reference to the Reference section as follows:

53. Belenguer, A. M. A focus on chasing pharmaceutical poly a morphs to design better oral drug formulations. Chemical Science 16, 2480–2482 (2025).

Figure 5 – The principal component analysis is interesting, but I am not sure how compelling the results are. It seems the ability to distinguish different substances is most pronounced in the Raman region, and ULFR and Brillouin perform slightly worse. Is it possible to conduct a PCA analysis with *only Raman*

versus *all three spectra combined*, and compare the two, to see how much utility is added by measuring all three regions at once? This may provide strong justification for the utility of the system.

We thank the reviewer for the observation, and we apologize for the confusion. It was not the authors' intention to use PCA to distinguish between different substances. Rather, the goal is to resolve between different mixing ratios of two components across the full 0-100% concentration range. Importantly, the incorporation of Brillouin spectral data is not intended to enhance discrimination in a categorical sense, but to introduce complementary contrast that reflects elastic property variations across the mixture series. When combined with either ULFR or Raman data, the Brillouin contribution preserves a more extended and approximately monotonic concentration-dependent trend in the principal component scores. In contrast, the ULFR + Raman combination exhibits an earlier saturation of the PC1 trajectory around intermediate compositions (~50%). Thus, the inclusion of Brillouin information expands the concentration range over which systematic compositional variation is captured within a single principal component representation.

Actions taken - We performed the concatenated analysis suggested by reviewer (Figures R6 and R7, new Supplementary Figs. 12 and 13). The combined analysis demonstrates the added value of integrating Brillouin spectra with the other spectral regions. Specifically, combinations including Brillouin data exhibit a more extended and approximately monotonic concentration-dependent trend in the PC1 scores across the mixture series. In contrast, the ULFR + Raman combination shows an earlier saturation of the PC1 trajectory at intermediate compositions. The results have been added to Supplementary Fig. 12 and Supplementary Fig. 13.

We have added the following statement on page 20 of the results section:

"*We further performed concatenated spectral analysis by combining data from all spectral domains (Supplementary Fig. 12) and in pairwise combinations (Supplementary Fig. 13). The results clearly demonstrate the benefit of incorporating Brillouin data in the analysis. Combinations including Brillouin spectra exhibit a more extended and near-monotonic concentration-dependent trend in PC1, whereas the combination of just ULFR and Raman data shows earlier saturation at intermediate compositions.*"

We added the following statement on pages 31 and 32 of the methods section:

"*To perform the concatenated spectral analysis in* Supplementary Fig. 12 and Supplementary Fig. 13*, spectral data from each domain (Brillouin, ULFR, and Raman) were preprocessed independently, including baseline correction and mean centering. To prevent dominance of blocks with higher variable density or variance, Frobenius-norm block scaling (equal total variance normalization) was applied prior to concatenation. The normalized spectral blocks were then concatenated into a single matrix, and PCA was performed on the fused dataset without additional scaling. This approach ensured balanced contributions from each spectral domain while preserving the internal variance structure of each block.*"

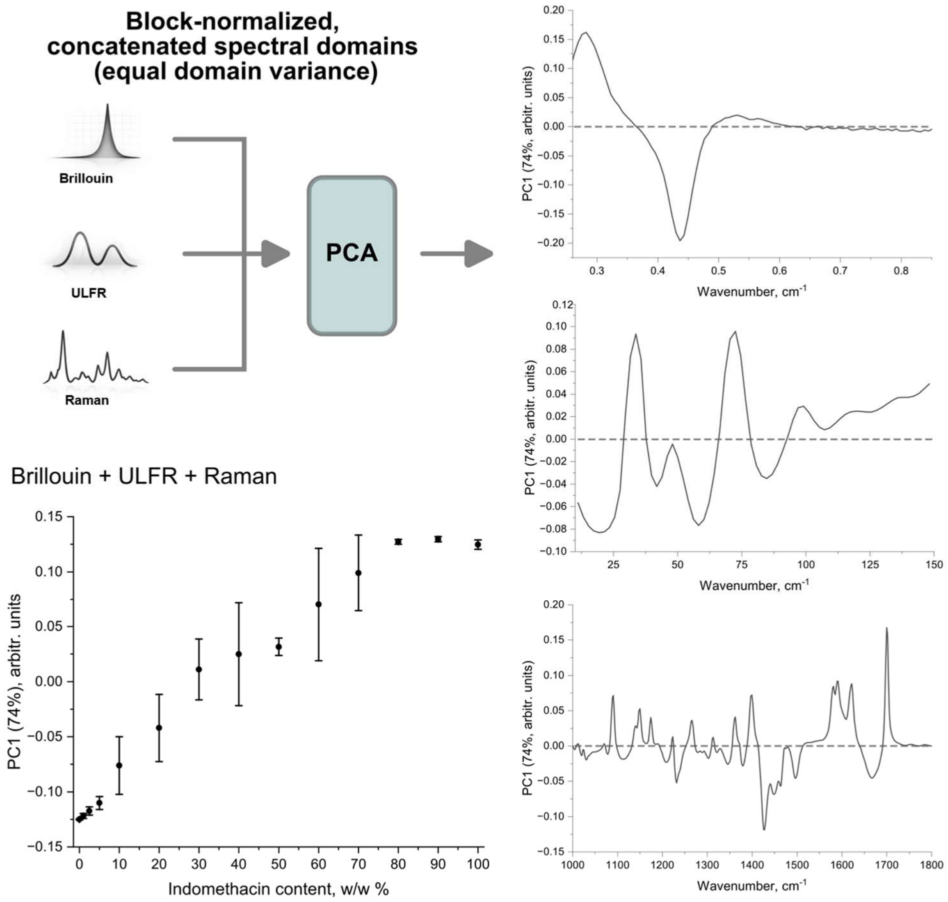

Figure R6 - PCA performed on block-normalized, concatenated spectral domains (Brillouin + ULFR + Raman) with equal variance contribution from each domain prior to fusion. The left panel shows the PC1 scores as a function of indomethacin content (w/w %), illustrating the concentration-dependent trend across the full mixture range. The right panels display the corresponding PC1 loadings for the Brillouin (top), ULFR (middle), and Raman (bottom) spectral regions, highlighting the relative contribution of each spectral domain to the first principal component.

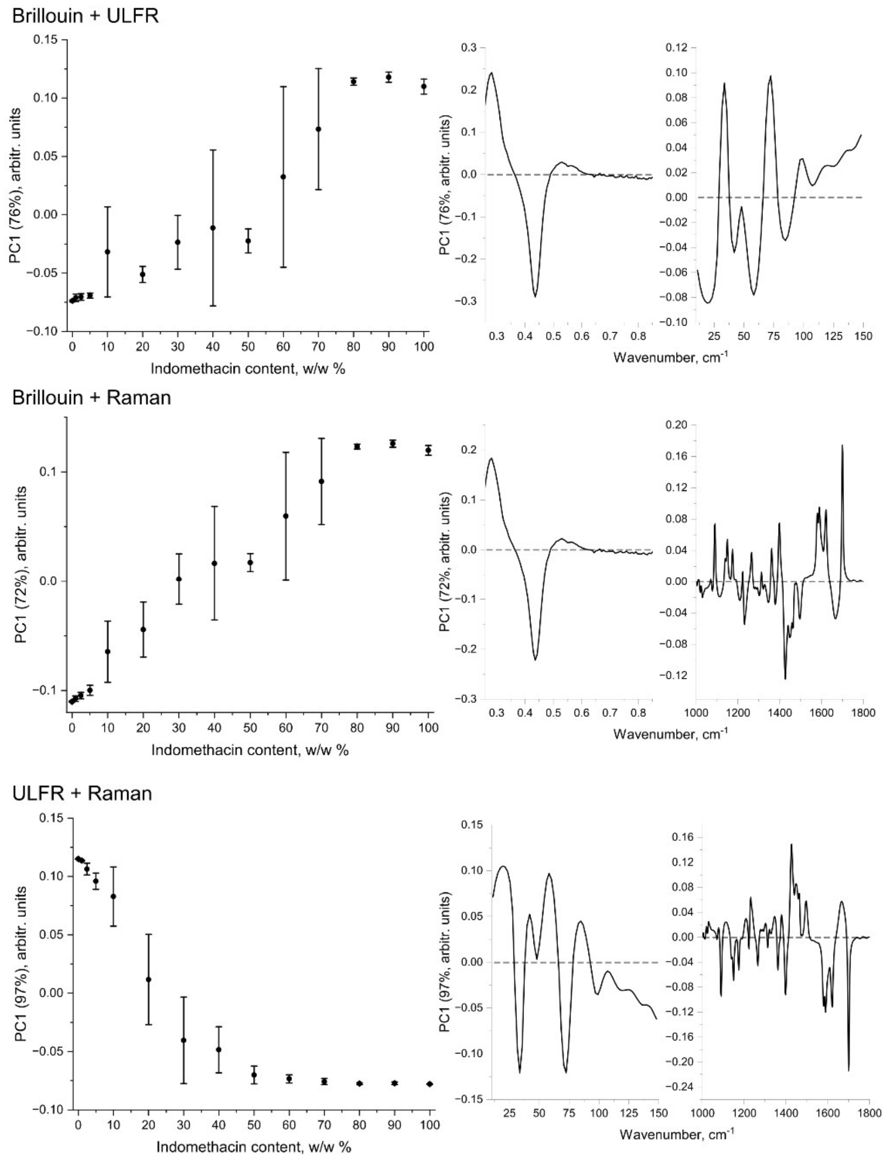

Figure R7 - PCA performed on block-normalized, concatenated spectral domains in pairwise combinations (Brillouin + ULFR, Brillouin + Raman, and ULFR + Raman), with equal variance contribution from each domain prior to fusion. In each row, the left panel shows PC1 scores as a function of indomethacin content (w/w %), while the right panels display the corresponding PC1 loadings for the respective spectral regions.

Figure 6 – The PCA maps appear largely similar to each other. It isn't clear to me how adding additional spectral regions is adding to the analysis of this sample. The significance of this measurement (combining all 3 regions) should be better justified, and if not significant, a better sample should be chosen.

The purpose of presenting similar PCA maps was to demonstrate that each of the three spectral regions is independently capable of identifying and distinguishing the API from the excipient, as well as in a concatenated modality. The apparent similarity between the maps reflects the robustness of the contrast provided by each spectral window. However, the individual maps do not exhibit perfect spatial overlap, which we attribute to mechanical and structural heterogeneity within the sample, even for a single chemical compound. To further investigate these subtle discrepancies, we performed additional analysis using the N-FINDR algorithm, enabling identification of heterogeneous features and providing a clearer interpretation of the minor mismatches observed between the spectral maps.

> **Actions taken** – To increase the clarity on the significance and purpose of our analysis, we have added the following statement on page 21 of the results section:
>
> "*The similarity between the individual PCA maps is expected, given that each spectral domain responds to the presence of a specific material. This validation is important because it allows us to confirm that each spectral domain can distinguish Ibuprofen from starch. However, despite the overall similarity, there are subtle differences among the maps. These differences mainly occur at the border of the region indicating the presence of Ibuprofen, leading to slight variations in the observed pattern. To better understand the subtle differences between the PCA maps, we performed further analysis using the N-FINDR algorithm.*"

Supp Figure 2 – The caption says "detected" but this is simulated data. Also, it would be useful to divide the resulting spectrum by the original to assess the consistency to the 3 dB reduction. I recommend doing this with real (rather than simulated) data as mentioned above, though the simulated data is fine to include to demonstrate the principle.

We agree that the terminology in the caption should be clarified, as the data shown are simulated rather than experimentally detected. In addition, to provide a quantitative assessment of the loss, we have performed the requested analysis using experimental data and present the resulting intensity ratio in Figure R4, thereby complementing the simulated results that illustrate the underlying principle.

> **Actions taken** - The legend has been updated to "*Simulated spectrum for 1.1 cm^{-1} resolution" and "Simulated spectrum for 1.3 cm^{-1} resolution*" in Supplementary Fig. 2 (Figure R8).
>
> The real data demonstrating the real measured loss has been reported in Figure R4 (Supplementary Fig. 8).

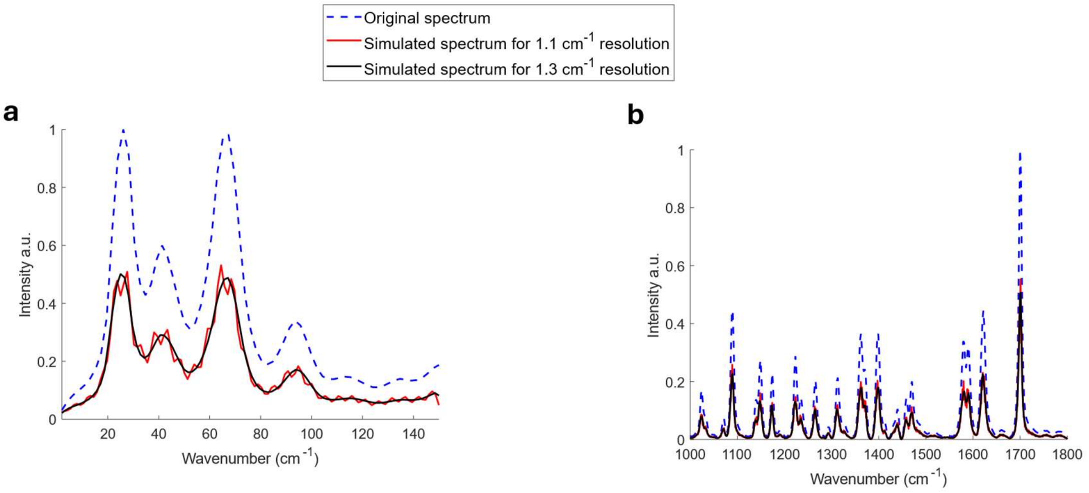

Figure R8 - Simulation of the modulation effect of the BIPD filter on (a) ULFR and (b) mid-frequency Raman spectra. A reference spectrum, measured using a commercial spectrometer, was interpolated to achieve a high number of data points (1,000,000 points; blue plot) and then multiplied by the BIPD filter's modulation function. The resulting modulated signal was integrated over intervals corresponding to the spectral resolution of the spectrometer (1.1 and 1.3 cm^{-1} in this case). Given the measured free spectral range (FSR) of 18.9 ± 0.5 GHz (~0.63 cm^{-1}) for the BIPD, a spectral resolution of ~33 GHz (1.1 cm^{-1}; less than twice the FSR) leads to a distortion in the spectral shape (red plot). In contrast, a resolution of ~39 GHz (1.3cm^{-1}; greater than twice the FSR) preserves the spectral information, resulting only in a ~50% reduction in signal intensity without introducing additional modulation effects (dark plot).

Minor concerns

Page 3 – name the variable vB for those unfamiliar.

We thank the reviewer for pointing this out.

> **Actions taken** – We have added "*where v_B is the Brillouin frequency shift*" on page 3 of the introduction section.

Page 6 – "these filters lack the bandwidth to isolate Brillouin/ULFR from elastic background". The purpose of these filters is to isolate Brillouin from Raman, and removal of the elastic peak is generally accomplished in some other way. This phrasing is a bit confusing to me and seems to conflate two issues.

We did not intend to conflate the separation of Brillouin and Raman signals with elastic peak suppression. Rather, our intention was to convey that, although such filters are effective in separating Brillouin from high-frequency Raman signals, they are not designed to function as dedicated filtering units for Brillouin and ULFR measurements, particularly they do not offer the capability to isolate these weak inelastic components from the strong elastic background. We have revised the text to clarify this distinction.

> **Actions taken** – We have replaced the sentence "*these filters lack the bandwidth to isolate Brillouin/ULFR from elastic background*" with "*the purpose of such filters is not to provide simultaneous elastic background removal for Brillouin or ULFR spectroscopy.*" on page 6 of the introduction section.

Page 5 – "…these spectral regimes may offer more valuable insights to enhance the precision…" This sentence is a bit vague.

We agree that the original sentence was vague. To improve clarity, we have revised the text to explicitly specify the intended applications, which are discussed in detail later in the manuscript.

> **Actions taken** – We have added the phrase "*to differentiate mixture percentages as well as sample preparation history to characterize*" on page 5 of the introduction section.

Page 6, Reference 32. There were earlier implementations of combined Raman and Brillouin, e.g. DOI: 10.1021/acs.analchem.5b02104

We thank the reviewer for the suggested reference.

> **Actions taken** – We have added the suggested references to the reference section as follows:
>
> *32. Traverso, A. J. et al. Dual raman-brillouin microscope for chemical and mechanical characterization and imaging. Analytical chemistry 87, 7519–7523 (2015).*

Page 6 – "lockin" -> "locking

We apologize for the typo.

> **Actions taken** – The typo has been corrected on page 6 of the introduction section.

The inset in Figure 2C is helpful. A similar inset showing modulation in the ULFR regime would be useful.

We agree that adding a similar inset illustrating modulation in the ULFR regime would improve clarity and provide a more complete understanding of the underlying principle.

> **Actions taken** – Figure 2c has been updated (Figure R1c) to include an additional inset illustrating the high-frequency modulation in the ULFR regime.

Fig 2 – Rayliegh *photons are* polarized

We thank the reviewer for pointing this out.

> **Actions taken** – We have added the phrase "*photons are*" in the caption of Figure 2 (Figure R1).

Page 9. The measured spectral resolution is 1 GHz. Is this sufficient for most Brillouin applications? Can this be improved in this study by deconvolving with the instrument response function, as is commonly done in Brillouin studies?

The reviewer raises an important point. While higher spectral resolution is often preferred in biological Brillouin applications, a resolution of approximately 1 GHz was sufficient for the objectives of our study. The spectral resolution can be readily improved by using a VIPA with a smaller free spectral range than the 60 GHz design employed here, and by selecting an appropriate FSR for the expected Brillouin frequency shift of the sample. Spectral fitting and deconvolution can also be used to effectively exceed the nominal resolution; however, Lorentzian fitting of the Brillouin peaks in our experiments was sufficient to analyze the frequency shift differences between samples.

Page 10. "Convert" – a better word might be "calibrate"

We thank the reviewer for the correct observation.

Actions taken – We have replaced the term "*convert*" with "*calibrate*" On page 13 of the results section.

Page 14 – Ball-milled, crystalline, and MQ should be introduced and better explained – as a reader it is not clear why these distinctions are important.

We agree that the terms ball-milled, crystalline, and MQ should be introduced more clearly and their relevance explained to help readers understand the importance of these distinctions in the context of our study.

Actions taken – We have added the following clarification of these samples on page 16 of the results section:

"*Crystalline γ-indomethacin, MQ amorphous indomethacin, and ball-milled amorphous indomethacin were selected to represent distinct solid-state conditions of the same compound. While both MQ and ball-milled samples lack long-range order, they arise from fundamentally different preparation routes, which can lead to differences in local packing and molecular mobility. This distinction is relevant in the broader context of amorphous solid dispersions (ASDs), which are now widely used formulation strategies and are produced through diverse processing routes, including melt-based and mechanically driven approaches. As different manufacturing pathways can yield amorphous materials with distinct physical properties, the ability to differentiate processing-dependent amorphous states is important for formulation development, stability assessment, and regulatory characterization*[54]."

We have added a new reference to the reference section as follows:

54. Moseson, D. E., Tran, T. B., Karunakaran, B., Ambardekar, R. & Hiew, T. N. Trends in amorphous solid dispersion drug products approved by the us food and drug administration between 2012 and 2023. International journal of pharmaceutics: X 7, 100259 (2024).

Page 18 – Recommend introducing the term "endmembers" in greater detail.

We agree with the reviewer that introducing endmembers can be useful for the reader.

Actions taken – We have added a detailed explanation of endmembers on page 21 of the results section as follows:

"*In the N-FINDR algorithm, endmembers are selected directly from the hyperspectral dataset as spectra associated with individual image pixels. Specifically, the method identifies the set of pixel spectra that maximizes the volume of the simplex enclosing the data in spectral space, such that the resulting endmembers correspond to real measured spectra rather than synthetic or averaged components*[55]."

Figure 6(b,c) – this is showing PC2 – should this be PC1?

While Figure 6a corresponds to PC1, Figure 6b and Figure 6c correspond to PC2. This choice reflects the presence of the relevant spectral features in different principal components.

Actions taken – To improve the clarity of the principal component loading plots, we have added the measured spectrum of ibuprofen as dashed lines in Figures 6a–c (Figure R9a-c).
The phrase "*Dashed lines represent measured spectral data of ibuprofen.*" has been added to the caption of Figure 6 (Figure R9).

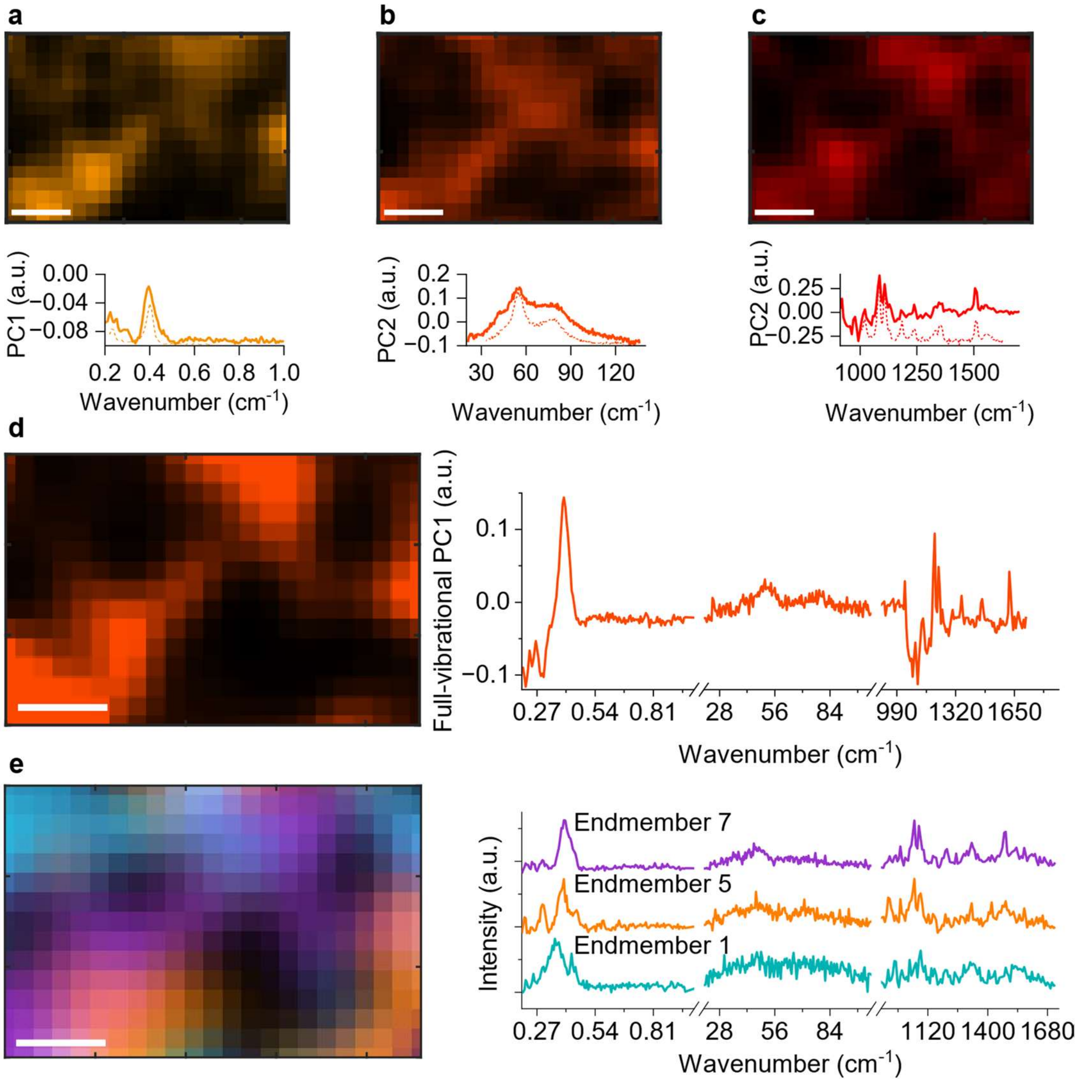

Figure R9 - Full-vibrational spectral maps of an ibuprofen tablet. ***a–c*** *Brillouin, ULFR, and Raman maps with respective loading plots associated with the relevant principal component (PC) show the mechanical, structural and chemical distribution of ibuprofen across the same field of view of a reference tablet. Dashed lines represent measured spectral data of Ibuprofen.* ***d*** *Concatenated full vibrational spectrum analyzed with PCA exhibits spectral features through the entire domain, further enhancing the image contrast and compound specificity.* ***e*** *Endmembers identified using the N-FINDR algorithm revealed mechanical heterogeneities of ibuprofen across chemically homogeneous areas. Scale bar corresponds to 100 µm.*

Other principal components (Figure R10, new Supplementary Fig. 14) have been added to the Supplementary Information. We have also added the statement "*Other PCs are reported in Supplementary Fig. 14.*" On page 20 of the results section.

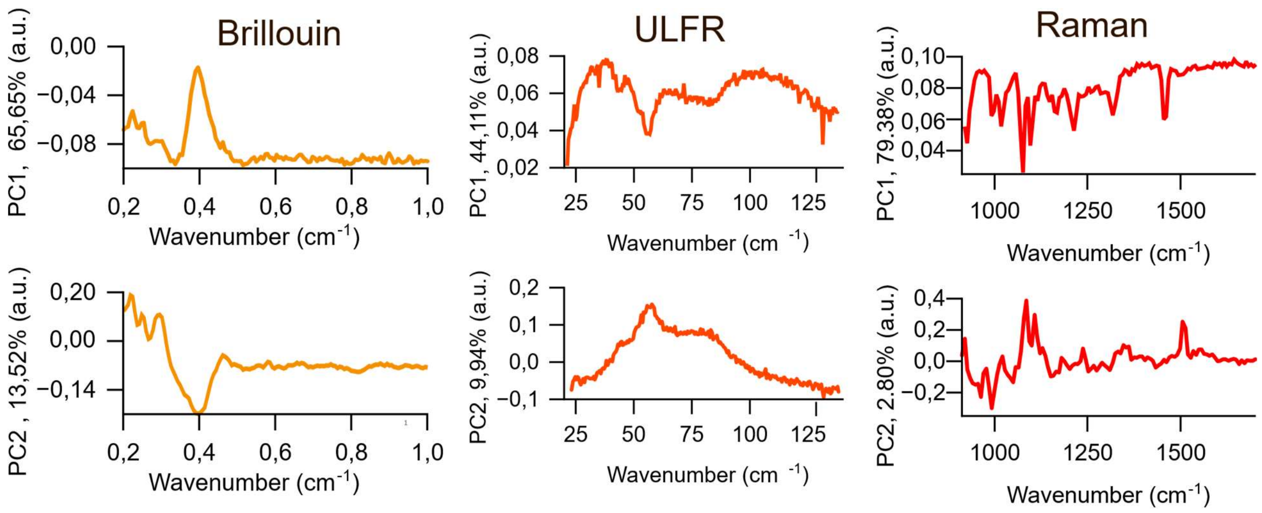

Figure R10 – Loading plots of PC1 and PC2 for Brillouin, ULFR, and Raman spectral regions along with their percentages are reported. The PCA maps in Figure 6 are plotted using PC1 of Brillouin and PC2 of ULFR and Raman spectrum and the reason for this choice is the presence of real spectral features related to Ibuprofen in these PCs (as shown in Figure 6). PC2 in Brillouin region is more representative of a pixel which lacks a peak related to Ibuprofen thus indicating the absence of Ibuprofen, furthermore a higher spectral component at lower frequencies is related to the starch content. PC1 in ULFR, is demonstrating a representative spectrum which lacks the features of a real ULFR spectrum of Ibuprofen and high intensity at lower frequencies is related to the broad vibrational density of states (VDOS) feature which is attributed to starch. PC1 in Raman region represents features that are related to the slightly shifted peaks of starch and ibuprofen coming closer to each other thus making PC1 more representative of the starch content.

Page 21 – "Our finding show…"
A discussion of the relevance of this technology to applications in biology would be welcome.

Indeed, numerous potential biological applications of this technology have been reported in the literature, and we agree that discussing their relevance would strengthen the manuscript.

Actions taken – We have added the following discussion on page 26 of the discussion section:

"*Recent studies have demonstrated that vibrational spectroscopy can provide complementary insights into nuclear organization in living cells. Low-frequency Raman imaging has been shown to reveal information about nucleic acid packing and ordering, enabling visualization of DNA compaction in the nucleus without labeling[58]. In parallel, combined Raman–Brillouin microscopy has been used to quantify both the biochemical composition and viscoelastic properties of chromatin, revealing differences between euchromatin and heterochromatin and highlighting the role of molecular constituents such as lipids in determining chromatin mechanics[59]. In this context, a system capable of simultaneously measuring Brillouin, ULFR, and Raman spectra could provide complementary mechanical, structural, and chemical information within the same measurement. Such multimodal characterization may therefore offer a useful approach for studying chromatin organization and nuclear architecture in living cells by linking molecular composition, structural ordering, and mechanical properties within a unified experimental framework.*"

We have added new references to the reference section as follows:

58. Machida, M. et al. Lipids are involved in heterochromatin condensation: A quantitative raman and brillouin microscopy study. bioRxiv 2025–02 (2025).

59. Trubitsyn, A. A. et al. Low-frequency label-free raman imaging of cells. The Journal of Physical Chemistry A 129, 11447–11453 (2025).

Supp Figure 1 – A legend explaining line colors would be helpful to aid the reader.

We agree that adding a legend explaining the color lines can aid the reader.

Actions taken – The legend explaining the line colors has been added to Supplementary Fig. 1 (Figure R11) to aid the reader.

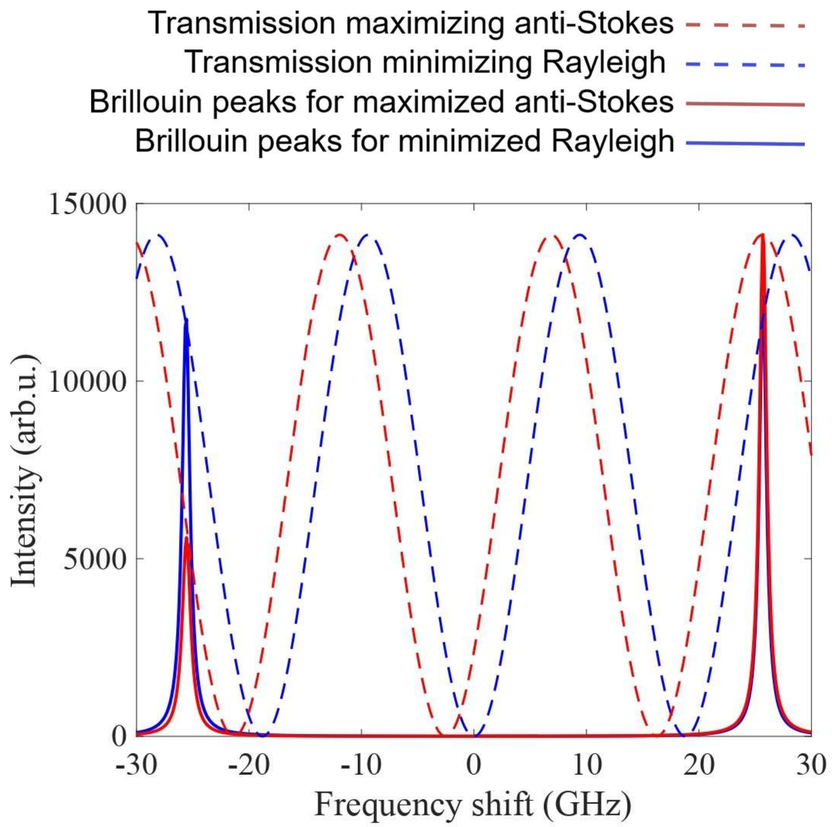

Figure R11 – Characterization of the FSR. We acquired calibrated Brillouin spectra coupling laser light into a standard optical fiber, whose spectrum featured strong Stokes and anti-Stokes Brillouin peaks from the core silica shifted by approximately 25 GHz from the central Rayleigh peak. The BIPD filter was first aligned to achieve minimum transmission at the laser (Rayleigh) frequency (dashed blue curve) and then adjusted to provide maximum transmission at the anti-Stokes Brillouin peak (dashed red curve). The reconstructed sinusoidal transfer function yielded a measured FSR of 18.9 ± 0.2 GHz (~0.63 cm $^{-1}$*), corresponding to an estimated birefringence of Δn = 0.26 ± 0.02. This value is in good agreement with the nominal birefringence of* YVO_4 *at 660 nm.*

Supp Figure 2 – The legend and caption have different numbers for resolution.

We appreciate the reviewer for noticing this issue.

> **Actions taken** – We have double checked the reported values and updated the resolution values in the caption of Supplementary Fig. 2 (Figure R8) as follows;
>
> "*Simulation of the modulation effect of the BIPD filter on (a) ULFR and (b) mid-frequency Raman spectra. A reference spectrum, measured using a commercial spectrometer, was interpolated to achieve a high number of data points (1,000,000 points; blue plot) and then multiplied by the BIPD filter's modulation function. The resulting modulated signal was integrated over intervals corresponding to the spectral resolution of the spectrometer (1.1 and 1.3 cm^{-1} in this case). Given the measured free spectral range (FSR) of 18.9 ± 0.5 GHz (~0.63 cm^{-1}) for the BIPD, a spectral resolution of ~33 GHz (1.1 cm^{-1}; less than twice the FSR) leads to a distortion in the spectral shape (red plot). In contrast, a resolution of ~39 GHz (1.3cm^{-1}; greater than twice the FSR) preserves the spectral information, resulting only in a ~50% reduction in signal intensity without introducing additional modulation effects (dark plot).*"

Supp Figure 5(a,b). These lines closely overlap. Are there error bars on these measurements? If not, it is difficult to claim a clear distinction between the two.

We agree with the reviewer that strong claims of differentiation based solely on the Raman data would not be appropriate, as Supplementary Fig. 9a and Supplementary Fig. 9b present only representative spectra without error bars or statistical analysis. In this form, the spectra of the differently prepared amorphous samples largely overlap, and any observed differences should therefore be interpreted cautiously. The Raman data in this context indicates only subtle variations rather than unambiguous separation. This limited contrast in the Raman domain further emphasizes the value of the Brillouin measurements, where statistically significant differences in frequency shift and linewidth are observed, providing clearer differentiation between the processing-dependent amorphous states.
